# Immuno-oncologic profiling of pediatric brain tumors reveals major clinical significance of the tumor immune microenvironment

**DOI:** 10.1038/s41467-024-49595-1

**Published:** 2024-07-10

**Authors:** Adrian B. Levine, Liana Nobre, Anirban Das, Scott Milos, Vanessa Bianchi, Monique Johnson, Nicholas R. Fernandez, Lucie Stengs, Scott Ryall, Michelle Ku, Mansuba Rana, Benjamin Laxer, Javal Sheth, Stefanie-Grace Sbergio, Ivana Fedoráková, Vijay Ramaswamy, Julie Bennett, Robert Siddaway, Uri Tabori, Cynthia Hawkins

**Affiliations:** 1 Arthur and Sonia Labatt Brain Tumour Research Centre, The Hospital for Sick Children, Toronto, ON Canada; 2 Department of Laboratory Medicine and Pathobiology, University of Toronto, Toronto, ON Canada; 3 Department of Pediatric Laboratory Medicine, The Hospital for Sick Children, Toronto, ON Canada; 4 Clinician Investigator Program, University of British Columbia, Vancouver, BC Canada; 5 Institute of Medical Sciences, University of Toronto, Toronto, ON Canada; 6 Department of Paediatrics, University of Alberta, Edmonton, AB Canada; 7 Neuro-Oncology Unit, Division of Haematology Oncology, The Hospital for Sick Children, Toronto, ON Canada; 8 Clinic of Pediatric Oncology and Hematology, University Children’s Hospital, Banská Bystrica, Slovakia; 9 Division of Medical Oncology and Hematology, Princess Margaret Cancer Centre, Toronto, ON Canada

**Keywords:** CNS cancer, Paediatric cancer, Cancer microenvironment, Cancer genomics

## Abstract

With the success of immunotherapy in cancer, understanding the tumor immune microenvironment (TIME) has become increasingly important; however in pediatric brain tumors this remains poorly characterized. Accordingly, we developed a clinical immune-oncology gene expression assay and used it to profile a diverse range of 1382 samples with detailed clinical and molecular annotation. In low-grade gliomas we identify distinct patterns of immune activation with prognostic significance in *BRAF* V600E-mutant tumors. In high-grade gliomas, we observe immune activation and T-cell infiltrates in tumors that have historically been considered immune cold, as well as genomic correlates of inflammation levels. In mismatch repair deficient high-grade gliomas, we find that high tumor inflammation signature is a significant predictor of response to immune checkpoint inhibition, and demonstrate the potential for multimodal biomarkers to improve treatment stratification. Importantly, while overall patterns of immune activation are observed for histologically and genetically defined tumor types, there is significant variability within each entity, indicating that the TIME must be evaluated as an independent feature from diagnosis. In sum, in addition to the histology and molecular profile, this work underscores the importance of reporting on the TIME as an essential axis of cancer diagnosis in the era of personalized medicine.

## Abstract (web summary)

The tumour immune microenvironment remains poorly characterised in paediatric brain tumours. Here, the authors develop a clinical immune-oncology gene expression assay to profile 1382 paediatric brain tumour samples, and reveal immune profiles and biomarkers that can contribute to diagnosis, prognosis, and treatment response.

## Introduction

Understanding the tumor immune microenvironment (TIME) is increasingly important in cancer biology and for patient management. The TIME has a major role in determining tumor subgroups, prognostic and predictive values, and is key for guiding therapy selection in many disease types, such as melanoma and colorectal cancer^1,2^. As a result, in many adult solid tumors, the TIME has been integrated into the analysis and determination of immune-based therapeutic approaches^3–5^. Pediatric brain tumors are the most common solid tumors in this age group. Although our understanding of initiating events, cell of origin, and mechanisms of tumor progression has significantly increased in the last two decades, the TIME has been relatively understudied in these cancers and the extent of immune activation across each tumor type is largely unknown^6^. Importantly, there are numerous immunotherapies in development that target different aspects of the TIME; however, it is not known which targets will be most effective in pediatric brain tumors^6^. This is especially of interest as certain types of traditionally lethal tumors have shown dramatic responses and prolonged survival using immunotherapy approaches^7,8^.

In contrast to recent large-scale multi-modality studies characterizing the TIME in adult gliomas and other central nervous system (CNS) tumors^9–14^, studies thus far in pediatric tumors have been limited by small sample sizes, heterogeneous methodologies, and lack of immunotherapy treatment data. Reflecting the experience of pathologists, studies have consistently identified greater numbers of infiltrating myeloid cells and lymphocytes in low-grade gliomas (LGG) compared to high-grade gliomas (HGG)^15–18^. There are conflicting results regarding diffuse midline glioma (DMG), a highly lethal infiltrative glioma^19^, with some reporting an overall immunologically neutral environment^20,21^, and others suggesting some degree of inflammatory cell infiltration^22,23^. Additionally, deconvolution-based approaches^24^ applied to large datasets of methylation^25^ and RNA sequencing data^23^ have provided an approximation of the proportion of immune cell types in different pediatric tumors, but without detailed clinical or pathologic correlation.

Given the need to characterize the TIME to stratify pediatric brain tumor subgroups, provide prognostic tools, and enable therapeutic decisions, we developed a functional transcriptomic assay that would (1) provide insights into the immune activation in pediatric CNS tumors, (2) integrate well with the routine pathology workflow and work with our extensive tissue archives, and (3) include markers that would represent the breadth of upcoming immunotherapy agents^26,27^. In this study, we analyze 1382 pediatric brain tumors representing a broad range of histologies and genotypes. This provides the most comprehensive evaluation to date of the pediatric-specific CNS TIME, which encompasses a diverse range of tumor types with detailed clinical and treatment outcomes. We identify important therapeutic and prognostic implications that confirm the importance of assessing the TIME routinely in these tumors as part of their diagnostic workup and pathologic reporting.

## Results

### Development of a clinical assay for immuno-oncologic profiling of pediatric brain tumors

**Fig. 1:**
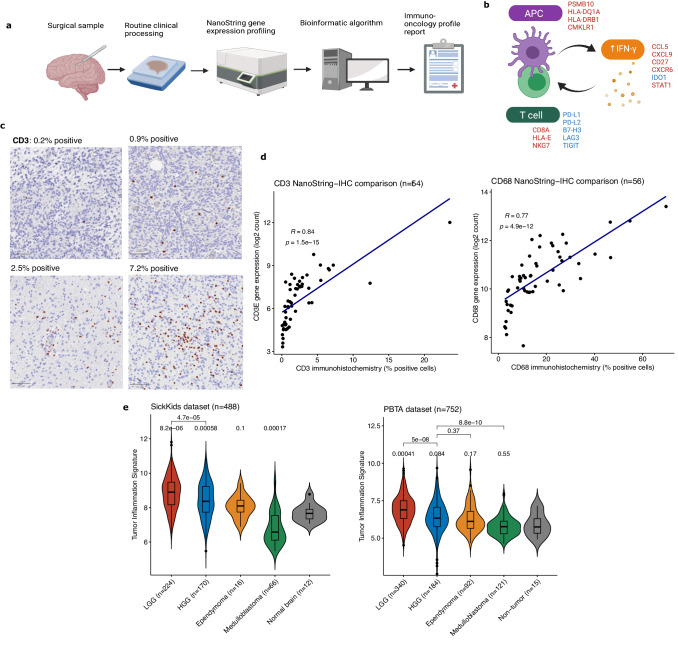
Overview of workflow, assay validation, and immune-oncologic profiling of pediatric brain tumors. **a** Schematic of immune-oncology profiling workflow using clinical tumor samples. Created with BioRender.com. (APC: antigen-presenting cell). **b** Diagram of 18 genes included in tumor inflammation signature (TIS) in relation to immune activation (red text denotes genes that increase immune activity and blue denotes genes that decrease immune activation). Created with BioRender.com. **c** Images of digital quantification of CD3 IHC. Representative areas of whole slide images were selected for visualization, with the percentage of positive cells in the entire slide indicated. Scale bar (bottom left) is 100 μm. Images taken at 100x magnification. **d** Scatter plots of correlation between gene expression by NanoString and percentage of positive cells by IHC for CD3 and CD8 in matched tissue sections. Correlation and *p*-values were calculated using the Spearman correlation. (**e**) Violin plots of TIS scores across major pediatric tumor types from SickKids and PBTA datasets, compared to non-tumor brain controls. Boxes show the median and interquartile range (IQR) of the data with whiskers extending to ±1.5 IQR. *p*-values calculated using two-tailed *T* test with Holm adjustment. (HGG: high-grade glioma; LGG: low-grade glioma).

To develop a clinically-validated functional genomic assay we designed a 103-gene immuno-oncology transcriptomic panel using the NanoString nCounter system, which can be applied to both fresh-frozen and FFPE tissue up to approximately 20 years old. To broadly characterize the immune response beyond our prior studies^7,8,28^ we included genes for specific cell types (e.g. *CD3E, CD8A, CD68, CD163, FOXP3, NKG7)*, immune checkpoints (PD-1/*PDCD1*, PD-L1/*CD274*, B7-H3/*CD276*, *TIGIT, LAG3, HAVCR2/*TIM3), additional therapeutic targets (*IDO1, CSF1R, CD47*, *NT5E*/CD73) and inflammatory pathways (cytotoxic T cell activity, JAK-STAT pathway, IFN-γ signaling, antigen presentation). We also incorporated the 18-gene tumor inflammation signature (TIS; see Methods for further details and Supplementary Data 4), a well-validated biomarker for immune checkpoint inhibitor (ICI) response that summarizes several components of the TIME in a single quantitative metric (Fig. 1a, b)^3,29,30^. The performance of the NanoString assay was rigorously validated through comparison with immunohistochemistry (IHC) on brain tumors and non-CNS validation tissue samples (Fig. 1c, d, S1; Supplementary Data 2). This analysis demonstrated excellent correlation between gene counts and the percentage of positive cells by IHC for CD3 (Spearman’s rank correlation=0.84, *p* = 1.5 × 10^−15^) and CD68 (Spearman correlation=0.77, *p* = 4.9 × 10^−12^), as well as good differentiation between gene expression levels for samples with PD-L1 IHC expression scored as high (>5% positive cells), low (1–5% positive), or none (<1%) (*p* = 0.003, Welch’s ANOVA; see Methods for more details regarding immunohistochemical quantification).

We tested 571 samples from our pathologically and clinically annotated institutional cohort (Supplementary Data 3) that represented the most common pediatric brain tumor types, including 224 low-grade gliomas (LGG), 170 high-grade gliomas (HGG), 16 ependymomas, 66 medulloblastomas (MB), and 12 non-tumor control brain samples (Fig. 1e; S2), plus an additional 83 mismatch repair deficient HGGs from the International Replication Repair Deficiency Consortium (IRRDC). To confirm our findings of the range of TIS scores across brain tumor subtypes, we used an external cohort of RNA sequencing for 811 samples from the Pediatric Brain Tumor Atlas (PBTA; Fig. 1e), which demonstrated similar findings overall.

Both LGG (median TIS = 8.86; *p* = 8.2 × 10^−6^) and HGG (median TIS = 8.52; *p* = 0.0006) had overall significantly more inflammation than non-tumor brain controls (median TIS = 7.66) (Fig. 1c). Ependymomas had lower levels of inflammation than LGG and HGG (median TIS = 8.09, *p* = 0.1), and had similar TIS score regardless of *ZFTA-RELA* fusion status (Fig S3a; *p* = 0.27 for fusion positive vs. negative). MB had less inflammation than other tumor types (median TIS = 6.58; *p* = 0.0002 vs. normal brain; Fig. 1c), although SHH-subgroup MB had higher inflammation than other MB subtypes (Fig S3b; median TIS = 7.72; *p* = 0.01 vs WNT, *p* = 0.007 vs Group 3, *p* = 0.004 vs. Group 4), which is consistent with several prior reports^25,31^. Importantly, in the PBTA dataset (Fig S3c), atypical teratoid/rhabdoid tumors (ATRT) had significantly higher TIS score (median TIS = 6.70) than non-tumor brain (median TIS = 5.75, *p* = 0.013), underscoring the potential for immunotherapy in these tumors, as has been previously reported^25,32^. While only a small number of cases were available, longitudinal analysis of 7 LGG and 6 HGG that were not treated with immunotherapy (Fig S3d) revealed that most (5/6) HGG had increases in their TIS over time (*p* = 0.044, paired Wilcoxon test) while this was not true for LGG (*p* = 0.76).

### Correlations of anti-tumor and pro-tumor components of immune response

**Fig. 2:**
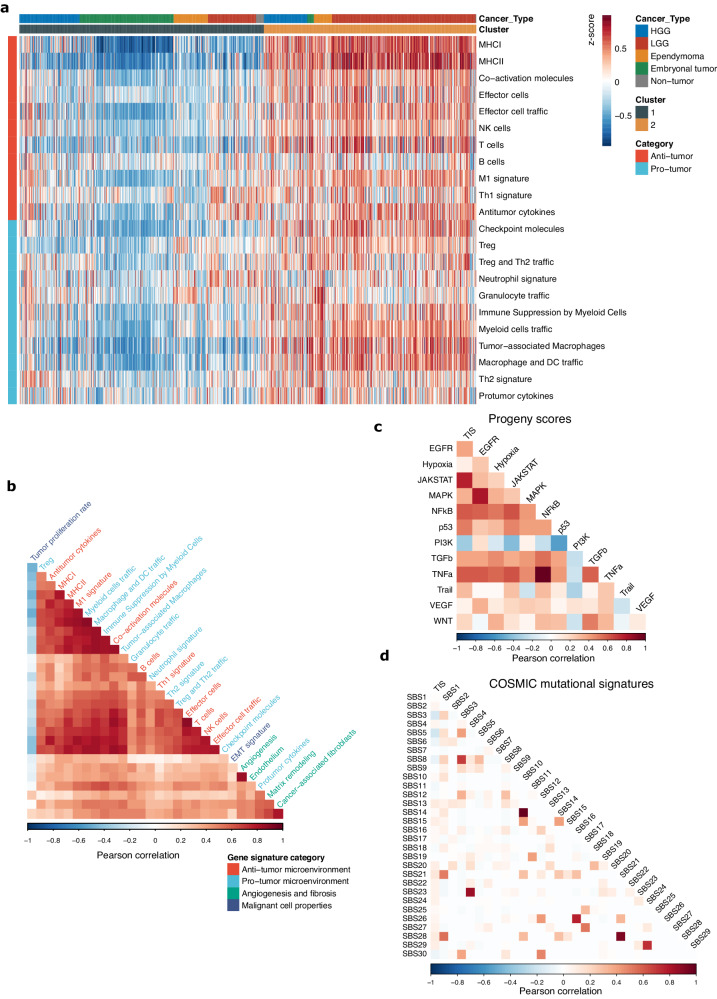
Patterns of immune regulation across pediatric brain tumors. **a** Heatmap of pan-cancer gene signatures of anti-tumor and pro-tumor immune processes from Bagaev et al. across 811 pediatric brain tumors from the PBTA. (HGG: high-grade glioma; LGG: low-grade glioma). **b** Pearson correlation matrix of pan-cancer immune gene expression signatures from Bagaev et al. **c**, **d** Pearson correlation matrix of TIS with (c) PROGENy cellular pathway activation scores and (**d**) COSMIC Single Base Substitution (SBS) mutational signatures.

To further investigate the patterns of immune response in pediatric brain tumors in conjunction with other cellular processes, we leveraged the matched WGS and RNA sequencing from the PBTA dataset (Supplementary Data 5). To evaluate the different components of the pro- and anti-tumor immune response, we applied previously described pan-cancer immune gene signatures (Supplementary Data 6)^5^. We found that pediatric brain tumors formed two clusters corresponding to immune-enriched and immune-desert phenotypes, with the former having high proportions of LGG and HGG, and the latter consisting of a greater number of ependymomas and embryonal tumors (Fig. 2a). Rather than discrete immunologic modules being independently regulated, as we had initially hypothesized, we observed a complex pattern in which anti-tumor and pro-tumor components of the immune response are broadly correlated and rise in unison (Fig. 2b). The finding that pro-tumor and anti-tumor aspects of the microenvironment are highly interrelated further supports the use of the TIS as a robust and clinically useful measure of multiple processes in immune regulation, encompassing T-cell activation, immune checkpoints, and antigen presentation.

We next applied the PROGENy^33^ cellular pathway scores to RNAseq data (Fig. 2c, S4a; Supplementary Data 7)) and found the TIS is most correlated with the JAK-STAT (Pearson correlation = 0.77, 0*p* < 2.2 × 10^−16^), TNFα (Pearson correlation = 0.61, *p* < 2.2 × 10^−16^), and NFkB signaling pathways (Pearson correlation=0.61, *p* < 2.2 × 10^−16^). Strikingly, TIS had an inverse correlation with the PI3K pathway activation (Pearson correlation = −0.36, *p* < 2.2 × 10^−16^), which is consistent with prior evidence implicating PTEN loss with immune evasion and immunotherapy resistance in multiple tumor types^34–36^. We also investigated associations between the TIS and COSMIC Single Base Substitution (SBS) signatures (Fig. 2d, S4b; Supplementary Data 8) and found that TIS is largely independent of mutational signatures in pediatric brain tumors, with the most notable finding being a weakly negative correlation with SBS3 (defective homologous recombination repair; Pearson correlation = −0.17, *p* = 1.5 × 10^−5^) and SBS5 (clock-like signature, Pearson correlation = −0.19, *p* = 8.6 × 10^−7^).

### High inflammation has prognostic significance in pediatric low-grade glioma

**Fig. 3:**
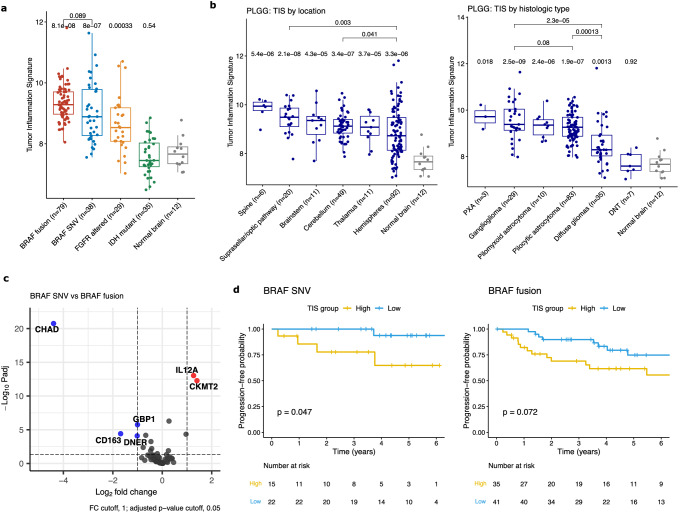
Overview and prognostic impact of TIME profiling in pediatric LGG. **a** Boxplot of TIS across genomic subtypes of LGGs. Boxes show the median and interquartile range (IQR) of the data with whiskers extending to ±1.5 IQR. *P*-values by two-tailed *T* test with Holm adjustment. **b** Boxplots of TIS scores for PLGG split by location and by histologic type. Boxes show the median and IQR with whiskers extending to ±1.5 IQR. *P*-values by two-tailed *T* test with Holm adjustment. (Diffuse glioma category includes diffuse astrocytomas and oligodendrogliomas; PXA: pleomorphic xantho-astrocytoma; DNT: dysembryoplastic neuroepithelial tumor). **c** Volcano plot of differentially expressed genes between PLGG with *BRAF* SNV (single nucleotide variant) and *BRAF* fusion. *P*-values by two-tailed *T* test with BH adjustment. **d** Kaplan–Meier curves of high vs low TIS scores in *BRAF*-mutant and *BRAF*-fused PLGG. *P*-values by log-rank test.

To study the TIME in LGGs, we profiled 224 tumors, covering the spectrum of driver alterations observed in childhood tumors, including *BRAF* fusions (*n* = 79), *BRAF* SNVs (*n* = 38; 35 with *BRAF* p.V600E, 1 each with V600ins, T599dup, and D594N; hereafter referred to as *BRAF* V600E samples for brevity), and *FGFR* alterations (29), as well as isocitrate dehydrogenase (*IDH*) 1/2-mutated LGG (*n* = 35; see Methods for more detail and Supplementary Data 9). Overall, the *IDH*-mutant LGG were immunologically cold (Fig. 3a; median TIS = 7.45, *p* = 0.54 vs. normal brain), which is consistent with prior studies on *IDH*-mutant gliomas^37,38^ and recent evidence demonstrating a mechanistic impact of the D-2-hydroxyglutarate oncometabolite on T-cell function^39^.

In contrast, many gliomas and glioneuronal tumors with pediatric-type LGG mutations had high levels of inflammation (Fig. 3a, S5a, b). Both *BRAF*-fused (median TIS 9.28, *p* = 8 × 10^−8^) and *BRAF* V600E (median TIS 8.88, *p* = 8 × 10^−7^) LGG, the two most common genomic alterations in pediatric-type LGG, had increased TIS vs non-tumor brain tissue, but did not have significant differences between each other (*p* = 0.09). *FGFR* altered LGG^40^, which activates the MAPK (mitogen-associated protein kinase) pathway upstream of *BRAF*, also had elevated inflammation (median TIS = 8.53, *p* = 0.0003 vs. normal brain). A similar trend was observed in the PBTA RNAseq cohort (Fig S5a), in which tumors with *BRAF* fusions (median TIS = 6.98; *p* = 3 × 10^−4^), *BRAF* V600E (median TIS = 7.34; *p* = 4.2 × 10^−6^), and non-canonical MAPK pathway alterations (median TIS = 7.18; *p* = 0.0024) had significantly higher inflammation than non-tumor brain (median TIS = 5.75). Although interestingly, in this dataset the *BRAF* V600E tumors had slightly more inflammation than *BRAF* fused tumors (*p* = 0.013).

While PLGG from all locations had higher TIS than normal brain, there were no major differences between tumor locations (Fig. 3b). Tumors in the suprasellar region (median TIS = 9.49; *p* = 0.003) and cerebellum (median TIS = 9.12; *p* = 0.04) had slightly higher inflammation than those in the cerebral hemispheres (median TIS = 8.73), however these differences were relatively small. Overall, this indicates that the inflammatory response is not driven by tumor location. When split by histologic diagnosis, circumscribed gliomas including pilocytic astrocytomas (median TIS = 9.26; *p* = 0.00013) and gangliogliomas (median TIS = 9.38; *p* = 2 × 10^−5^) both had substantially higher inflammation than diffuse gliomas (median TIS = 8.29). Overall, many LGG had upregulation of immune checkpoints, suggesting that ICI may be an effective strategy in tumors that are incompletely resected or recur (Fig S5c).

Differential expression analysis was performed comparing *BRAF* V600E with *BRAF* fused tumors (Fig. 3c; Supplementary Data 10) This demonstrated substantially higher expression of *IL12* (a pro-inflammatory cytokine) in *BRAF* V600E samples (log2FC = 1.3, adjusted *p*-value = 9 × 10^−14^), suggesting that these tumors may have a greater degree of T-cell activation, while in *BRAF* fused tumors *CD163* (a marker of anti-inflammatory or M2 macrophages) was higher (log2FC = −1.7, adjusted *p*-value = 4 × 10^−5^), indicating greater component of immunosuppressive macrophages. Interestingly, in some LGG groups, the level of inflammation had prognostic significance, which is consistent with prior studies from our group using methylome-based estimates of inflammation^28^. In *BRAF* V600E LGG, high TIS was associated with poorer progression-free survival (Fig. 3d; *p* = 0.047), while in *BRAF* fused LGG a similar trend was observed but did not reach statistical significance (*p* = 0.072).

### *BRAF* p.V600E mutant low-grade gliomas form distinct immunologic clusters

**Fig. 4:**
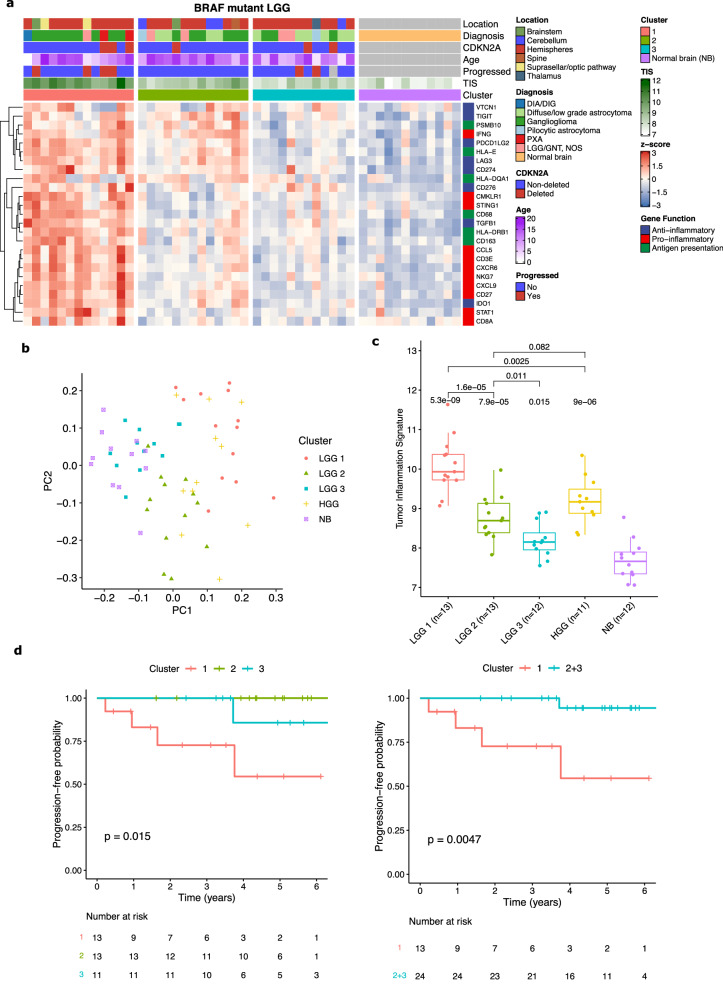
Clusters of immunologic response in *BRAF* V600E mutant LGG. **a** Heatmap of three distinct immunologic clusters of *BRAF* mutant LGG showing expression of selected genes of interest. (DIA/DIG: desmoplastic infantile astrocytoma/ganglioglioma; PXA: pleomorphic xantho-astrocytoma; LGG/GNT NOS: low-grade glioma/glioneuronal tumor, not otherwise specified; NB: normal brain). **b** PCA plot of three LGG clusters and *BRAF* V600E mutant PHGG compared to normal brain. **c** Boxplot of TIS scores for three LGG clusters and *BRAF* V600E mutant PHGG compared to normal brain. Boxes show the median and interquartile range (IQR) of the data with whiskers extending to ±1.5 IQR. *P*-values by two-tailed *T* test with Holm adjustment. **d** Kaplan-Meier curves of progression-free survival for the three LGG immune clusters and of cluster 1 compared to cluster 2 + 3. *P*-values by log-rank test.

Given the wide variability in TIS scores between tumors with the same genetic drivers, we were interested in further investigating the heterogeneity in immunologic responses to LGG. Unsupervised hierarchical clustering of LGG with *BRAF* V600E and other rare *BRAF* SNVs using 103 genes on the NanoString panel demonstrated three well-defined clusters corresponding to distinct patterns of immune response (Fig. 4a; Supplementary Data 11). Cluster 1 had a high level of inflammation with upregulation of virtually all the genes on the panel, including genes pertaining to both lymphocyte and myeloid cell regulation. Cluster 2 had an intermediate level of immune activation, with greater expression of APC-related compared to T-cell genes. Finally, cluster 3 had a near-normal level of immune activation, similar to non-tumor brain. The tumor content (TC; Fig S6a; Supplementary Data 12) as assessed by a neuropathologist was higher in cluster 1 (median TC = 60%; *p* = 0.01 vs cluster 2, *p* = 0.028 vs cluster 3) but not different between clusters 2 (median TC = 35%) and 3 (median TC = 40%; *p* = 0.52). Analogous analysis using *BRAF* fused PLGG did not yield well defined clusters, indicating that this is likely a more immunologically homogeneous group of tumors.

PCA projection of the three clusters further demonstrated that cluster 3 is the most similar to normal brain and cluster 1 is the least similar (Fig. 4b). There were significant differences in TIS scores between all three groups, with cluster 1 having the highest scores (median TIS = 9.93, *p* = 5.3 × 10^−9^ vs. normal brain; Fig. 4c), cluster 3 the lowest (median TIS = 8.15, *p* = 0.015 vs. normal brain), and cluster 2 an intermediate level (median TIS = 8.69, *p* = 7.9 × 10^−5^ vs. normal brain). For comparison, *BRAF* V600E mutant PHGG were mainly distributed near LGG cluster 1 on the UMAP plot and had intermediate TIS scores between cluster 1 and cluster 2 (median TIS = 9.17, *p* = 9 × 10^−6^ vs. normal brain; Fig. 4c). There was a prognostic difference between the clusters, with cluster 1 having substantially worse PFS than the other two clusters (Fig. 4d; *p* = 0.015 for 3-way comparison and *p* = 0.0047 for cluster 1 vs cluster 2 + 3). This remained significant when patients with gross total resections (GTR) were excluded (Fig S6b, *p* = 0.015) and in multivariate analysis (*p* = 0.041, Cox regression) correcting for other known prognostic factors, including *CDKN2A* deletion, age, histology, and location (Fig S6c, d; note that due to lack of progression events in patients with a GTR this variable could not be included in the Cox regression). This further reinforces the survival stratification by TIS score that we found in these tumors and the role of high inflammatory infiltrates as an important negative prognostic marker in *BRAF* mutant LGG.

### Pediatric high-grade gliomas have high immune activation and potential immunotherapeutic targets

**Fig. 5:**
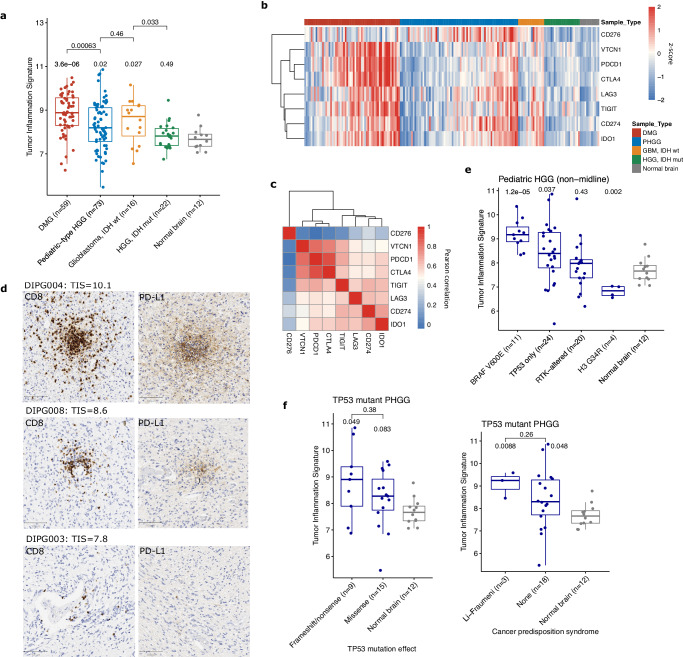
Elevated inflammation levels in pediatric-type HGG. **a** Boxplot of TIS across pediatric and adult-type HGGs. Boxes show the median and interquartile range (IQR) of the data with whiskers extending to ±1.5 IQR. *P*-values by two tailed *T* test with Holm adjustment. (DMG: diffuse midline glioma; IDH: isocitrate dehydrogenase). **b** Heatmap of gene expression of immune checkpoints and other inhibitory markers across HGG types. Values represent log2 gene counts scaled to mean = 0 and standard deviation = 1. (PHGG: pediatric-type HGG). **c** Heatmap of Pearson correlation between immune checkpoint gene counts on NanoString. **d** Representative images of CD8 and PD-L1 IHC from 3 diffuse midline gliomas. Scale bar (bottom left) is 100 μm. Images taken at ×100 magnification. **e** Boxplot comparing TIS scores for genetically defined subtypes of pediatric hemispheric HGG. Boxes show median and IQR with whiskers extending to ±1.5 IQR. *P*-values by two-sided *T* test with Holm adjustment. (RTK: receptor tyrosine kinase). **f** Boxplots for HGGs with only TP53 mutations identified, comparing protein-altering effect of mutation, and germline status of TP53 mutation. Boxes show median and IQR with whiskers extending to ±1.5 IQR. *P*-values by two-sided Wilcoxon rank sum test, unadjusted.

To investigate the TIME of HGGs across ages and locations we studied diffuse midline gliomas (DMG, *n* = 59), non-midline pediatric high-grade gliomas (PHGG, *n* = 73), and adolescent and young adult (AYA) HGG (patients aged 15–40 years), which included *IDH*-mutant (*n* = 22) and *IDH*-wildtype (*n* = 16) tumors (Supplementary Data 13). Both midline and hemispheric pediatric-type HGGs had a range of TIMEs, with high inflammation in many tumors (Fig. 5a), and elevated expression of targetable immune checkpoints (Fig. 5b, c, S6a). Interestingly, the expression levels of several immune checkpoints that are targetable with antibodies (*CTLA*, PD-1/*PCDC1*, PD-L1/*CD274*, *TIGIT*, *LAG3*) were highly correlated with each other (Fig. 5c), while *CD276*/B7-H3, a promising chimeric antigen receptor (CAR)-T cell target^32,41^ was complete uncorrelated, suggesting that distinct populations of patients may benefit from these two approaches.

In particular, DMGs (median TIS = 8.88; *p* = 3.6 × 10^−6^ vs. normal brain, *p* = 0.00063 vs. PHGG; Fig. 5a) had substantially higher inflammation than both the non-tumor brain (median TIS = 7.66) and groups of hemispheric HGG (median TIS = 8.19; *p* = 0.02 vs normal brain). Several tumors (5/58 in SickKids cohort; 9%) had very high TIS > 10 (Fig S7b), indicating that these tumors are not exclusively immunologically cold, as has been previously reported^19–21,42^. A similar trend was observed in the PBTA RNAseq dataset (Fig S7c), in which both DMG (median TIS = 6.27, *p* = 0.08 vs. non-tumor brain) and PHGG (median TIS = 6.51, *p* = 0.03) had elevated inflammation compared to non-tumor brain (median TIS = 5.75), and no significant difference between each other (*p* = 0.3). The AYA HGGs had a clear distinction by *IDH* mutational status (Fig. 5a). *IDH*-wildtype HGGs had similar TIS scores to the pediatric HGGs (median TIS = 8.71; *p* = 0.027 vs. normal brain, *p* = 0.46 vs. hemispheric PHGG), while the *IDH*-mutant HGG had inflammation that was comparable to normal brain (median TIS = 7.83; *p* = 0.49 vs. normal brain, *p* = 0.033 vs. *IDH*-wildtype HGG).

Given that significant inflammation has not been previously described in DMG, we verified the presence of inflammatory infiltrates with IHC for CD8, PD-L1, CD3, and CD68 (Fig. 5d, S8). As has been described, some tumors (DIPG127, DIPG005) have an immunologically cold TIME, however other samples demonstrate extensive T-cell infiltrates and even strong PD-L1 positivity (DIPG004, DIPG008). One explanation for this is that our study included substantially more DMG samples than prior ones (59 in-house and 99 PBTA compared to 13 samples in ref. ^21^ and 9 in ref. ^20^), which potentially allowed us to capture a broader range of immunologic states in these tumors than had previously been appreciated. To study the impact of treatment course on the DMG TIME, using the PBTA RNAseq dataset we compared TIS scores for DMGs from primary tumor samples (*n* = 65, median TIS = 6.38) with those from tumor progression/recurrence (*n* = 34, median TIS = 6.58), and found no difference in scores (*p* = 0.85; Fig S7d; Supplementary Data 14).

While DMGs are predominantly driven by H3 K27M mutations, the non-midline PHGGs had a more diverse range of alterations. These tumors had highly variable inflammation, with some patients having among the highest TIS scores in our entire cohort (>10), while others were immune cold, with lower TIS than the non-tumor brain. To further understand this variability, we studied the relationship of TIS with driver mutations and other genomic features (Fig. 5e, f). *BRAF* V600E mutant HGG had the highest inflammation levels among PHGG (median TIS = 9.17; *p* = 1.2 × 10^−5^ vs. normal brain), which is consistent with the known immunogenicity of the *BRAF* V600E neoantigen in other cancers^43,44^. RTK-altered HGG, which mainly consisted of *EGFR* and *PDGFRA* alterations (median TIS = 7.98, *p* = 0.4) had similar inflammation levels to normal brain (median TIS = 7.66), and a small number of H3.3 G34R mutant tumors had significantly lower inflammation (median TIS = 6.84, *p* = 0.002 vs. normal brain). Using PBTA WGS data (Supplementary Data 15, 16), we found a weak correlation between TIS and total number of missense (Fig S9; *R* = 0.24, *p* = 0.016) and nonsense (*R* = 0.24, *p* = 0.013) mutations, and no relationship between number of frameshift indels and copy number alterations (including overall number, gains, and losses).

There were 24 PHGGs where the only identified driver mutation was in *TP53*, which had highly variable TIS scores (Fig. 5e, f; median TIS = 8.39, *p* = 0.037 vs. normal brain). We identified a relationship related to the protein-coding effect of the *TP53* mutation, consistent with evidence suggesting higher neoantigenicity of frameshift compared to missense mutations^45^. Tumors with frameshift or nonsense mutations in *TP53* (median TIS = 8.90; *p* = 0.049 vs. normal brain, *p* = 0.38 vs *TP53* missense, Wilcoxon test) had higher TIS than those with missense *TP53* mutations (median TIS = 8.27, *p* = 0.08 vs. normal brain). Interestingly tumors from 3 Li-Fraumeni syndrome (LFS) patients (median TIS = 9.24; *p* = 0.009 vs. normal brain, Wilcoxon test), had higher inflammation than most somatic *TP53*-mutant HGG (median TIS = 8.3, *p* = 0.05 vs. normal brain, *p* = 0.26 vs LFS patients). While this requires further investigation in a larger number of patients, this finding potentially expands the range of cancer predisposition syndromes with an impact on the TIME in CNS tumors.

### High TIS predicts immunotherapy response in mismatch repair deficient HGGs

**Fig. 6:**
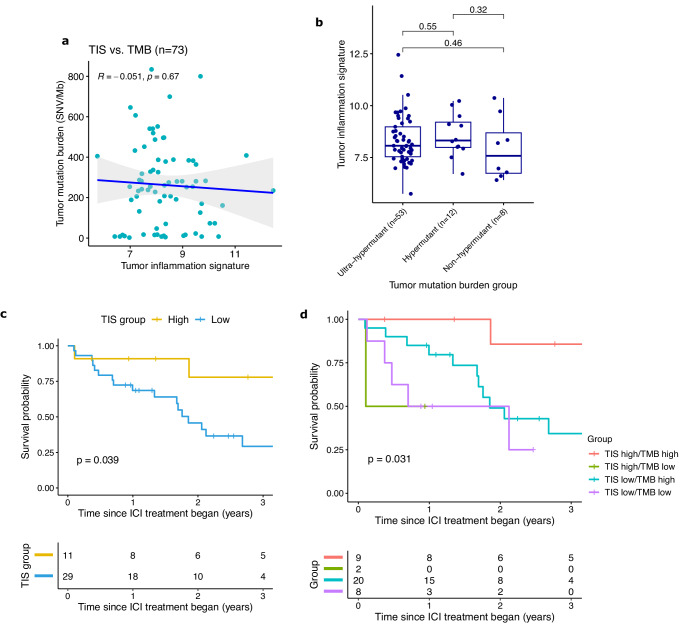
TIS as a biomarker for immunotherapy response in mismatch repair deficient HGG. **a** Scatterplot of tumor inflammation signature (TIS) vs. tumor mutation burden (TMB) in MMRD HGG. Correlation and *P*-values using Spearman correlation coefficient. Error bands represent the 95% confidence interval. **b** Boxplot of TIS split by TMB groups of ultra-hypermutant (TMB > 100 SNV/Mb), hypermutant (TMB 10-100), and non-hypermutant (TMB < 10). Boxes show the median and interquartile range (IQR) of the data with whiskers extending to ±1.5 IQR. *P*-values by two-tailed *T* test without adjustment. **c** Kaplan–Meier curves of overall survival for MMRD HGG treated with ICI, split by high (TIS > 9) and low inflammation. *P*-value by log-rank test. **d** Kaplan–Meier curves of overall survival for MMRD HGG treated with ICI, separated into 4 groups by TIS high/low status and TMB high/low status. *P*-value by log-rank test.

Expanding on our previous work demonstrating excellent response to ICI in mismatch repair deficient (MMRD) HGG^7,8^, we profiled 83 tissue samples from MMRD pediatric HGGs, 73 of which had matched whole exome sequencing (median TMB = 254 SNV/Mb; range 2-834; Supplementary Data 17–20). TIS and overall tumor mutation burden (TMB), as well as total numbers of different mutation types (Fig S10a), were completely uncorrelated in these patients (Fig. 6a; Spearman correlation = −0.051, *p* = 0.67), indicating that they provide complementary information for clinical decision making. Furthermore, when TMB was separated into ultra-hypermutant (TMB > 100 SNV/Mb), hypermutant (TMB 10-100), and non-hypermutant cases, there was no difference in TIS between the three groups (Fig. 6b). This finding, in addition to the weak correlation between total missense and nonsense mutations with TIS in non-MMRD PHGG (Fig S9), is in line with prior studies that have not found a consistent correlation between these features in other cancer types^4,46^. This demonstrates that, despite its utility in predicting ICI response^47,48^, TMB cannot be viewed as a surrogate for tumor inflammation.

Interestingly, a comparison of MMRD tumors with their MMR-proficient (MMRP) counterparts did not reveal an overall difference in their inflammation levels (Fig S10b). MMRD HGG (median TIS = 8.16; *p* = 0.0037 vs. normal brain, *p* = 0.76 vs. MMRP PHGG) had similar TIS scores to the cohort of pediatric hemispheric HGGs (median TIS = 8.19), which were both elevated compared with normal brain (median TIS = 7.66). Similar to what was observed in non-MMRD pediatric HGG, tumors with a truncating or frameshift TP53 mutation (median TIS = 8.67; Fig S10c) had higher TIS than those with a missense mutation (median TIS = 7.88; *p* = 0.008, Wilcoxon test).

Of the MMRD HGG samples we profiled, there 40 patients that had been treated with ICI (either nivolumab or ipilimumab) and had pre-treatment tissue available (Supplementary Data 18). While to date our patient selection of pediatric HGG for immunotherapy has been largely based on elevated tumor mutation burden (TMB), not all patients with very elevated TMB (i.e. >100) respond to ICI. We hypothesized that, in addition to high TMB, patients also required an inflammatory TIME, and accordingly were interested in developing multimodal biomarkers. Strikingly, MMRD HGG receiving PD-1 blockade with high TIS had significantly greater overall survival (OS) than low TIS tumors (*p* = 0.039; Fig. 6c, S11a), as did ultra-hypermutant tumors (TMB > 100 SNV/Mb) (Fig S11b; *p* = 0.03). Combining TIS and TMB revealed a subgroup of TIS-high/TMB-high tumors with OS of 86% at 3 years compared to 34% for tumors with TIS-low/TMB-low (*p* = 0.03; Fig. 6d, S11c). This dramatically illustrates the potential for the incorporation of multiple metrics to better predict immunotherapy response and optimize the selection of immune-based treatments versus conventional chemotherapy and radiation.

Furthermore, despite the excellent clinical response of many MMRD HGGs to immune checkpoint inhibitors^7,8^, these tumors in fact overall demonstrated only modest elevations of PD-1 and *CTLA4* gene counts compared to normal brain, and no significant difference in PD-L1 (Fig S12). There were more dramatic increases in the levels of *LAG3* and *CD276* (B7-H3), suggesting these alternate immune checkpoints may warrant further investigation as therapeutic targets^26,32,41^.

**Fig. 7:**
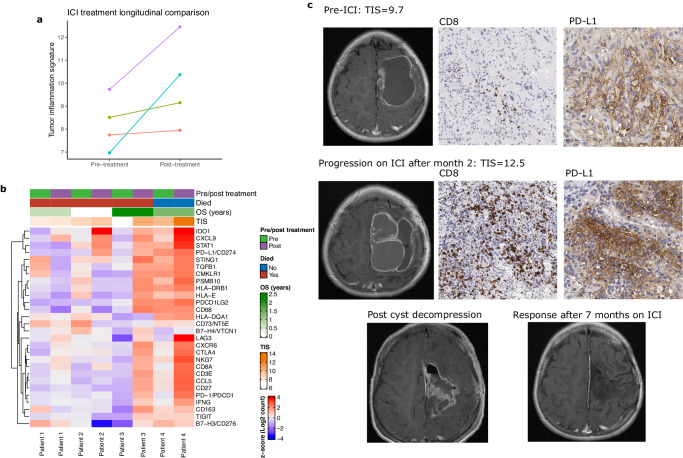
TIME alterations with immune checkpoint inhibition (ICI). **a** Scatter plot comparing longitudinal TIS scores pre- and post-ICI treatment in four MMRD HGGs with matched tissue samples. **b** Heatmap of NanoString counts for selected genes pre- and post-ICI treatment for four patients with longitudinal sampling. **c** Representative MRI and histology (CD8 and PD-L1 IHC) images of pre-ICI treatment and post-treatment samples from a patient with MMRD HGG. Scale bars (bottom left) are 100 μm for CD8 (images taken at ×100 magnification) and 50 μm for PD-L1 (images taken at ×200 magnification).

Comparison of matched pre- and post-ICI treatment samples in four patients with multiple surgeries demonstrated that all four had an increase in TIS following treatment (Fig. 7a), which confirms the expected effect of ICI in stimulating interferon-γ -related signaling and resulting immune activation^49,50^. Given the complexity of the immune response to these therapies and the small number of patients, it was not possible to identify a consistent well-defined pattern of TIME alterations following therapy (Fig. 7b). However, in general MMRD-HGG demonstrated an upregulation of one or more immunosuppressive factors, such as *IDO1* or alternate immune checkpoints (*LAG3* and/or *TIGIT*). In contrast, B7-H3 (*CD276*) gene levels declined in all patients.

IHC for CD8 and PD-L1 confirmed increases in inflammatory infiltrates in post-treatment samples (Fig. 7c, S13)—in some cases dramatically so, as in the example of a patient who had an excellent response to ICI and was alive at last follow up 16 months after the initiation of treatment (Fig. 7c). In this example, the patient remained on single agent immune checkpoint inhibition after repeat surgery, based on our profiling demonstrating dramatic increases in markers of immune activation in response to treatment. This illustrates the potential for longitudinal immunologic assessments to guide personalized treatment strategies, particularly whether the TIME is supportive enough to continue monotherapy for a given patient (as it was in this case), and if not, which combination therapy (e.g. anti-PD-1/PD-L1 in combination with anti-CTL4, anti-TIGIT, or anti-LAG3) is most likely to be effective.

## Discussion

In this study we have presented the immune-oncologic profiling of 1382 pediatric brain tumors through a clinical-grade gene expression assay and RNA-sequencing from the PBTA. Given the breadth of this dataset, we focused on the tumor inflammation signature (TIS)^3,29,30,51^ as a marker that integrates 18 genes comprising several aspects of the immune response into a single numerical value, providing a summary of the overall degree of immune activation in a sample. Within given tumor subtypes there is a wide range of immunologic states; our data shows this inflammatory environment to be clinically relevant to characterize across a spectrum of brain tumors and that this can be done in a flexible and clinically pragmatic workflow.

A key strength of our dataset is the inclusion of a wide range of tumor types, both histologically and genetically defined, extensive clinical and molecular annotation, and long-term follow up in most patients. In particular, our MMRD HGG dataset is the largest cohort of ICI-treated pediatric brain tumors published to date^8^ and the success in this group was a major advance in the use of immunotherapy for CNS tumors. Through the analysis of PBTA patients, we confirm that the overall findings from our in-house cases are broadly applicable and consistent in an external dataset using a different gene expression platform.

One limitation of this study is the use of gene expression as the primary modality for immune profiling, although this was validated through extensive matched IHC. A motivating factor for this study was our desire to make maximal use of our extensive FFPE archives of well annotated tissue samples from many rare tumor types, as well as to use this as a clinical tool, for which cost and integration within the pathology workflow are important factors. Currently high throughput protein expression assays are not sufficiently developed for clinical implementation, while multiplexed IHC is limited by high cost and the time requirements for analysis, making gene expression the most reliable and pragmatic platform for comprehensively evaluating the various processes in the TIME currently.

Our results in both LGG and HGG indicate that many pediatric gliomas have evidence of high immune activation, although to a greater degree and more consistently in low-grade compared to high-grade tumors, and in circumscribed over diffuse LGGs. In contrast, *IDH*-mutant tumors (both LGG and HGG) from our adolescent and young adult cohort have similar TIS scores to normal brain. Many DMGs and hemispheric PHGGs have high expression of several immune checkpoints, including those with currently available drugs (PD-1, PD-L1) and drugs under development (LAG3, TIGIT), which suggests that this is an unexplored treatment strategy that could benefit some of these patients. We anticipate that with further clinical experience, and the translation of novel immunotherapies, the number of pediatric LGG and HGG patients who can benefit from immune-based treatment (potentially in combination with other targeted agents) will expand in the future. In particular, *BRAF* p.V600E-mutant HGGs have significantly elevated TIS scores compared to other pediatric HGG, as well as a prior study showing increased T-cell infiltration in these tumors^52^, identifying this group of patients as of particular interest for immunotherapy.

In pediatric LGG, we have previously shown that *BRAF* mutant cases have worse outcomes than those with *BRAF* fusions, and that these patients benefit from targeted BRAF and MEK inhibition^53–56^. Our finding that a high TIS score is a negative prognostic marker in *BRAF* mutant tumors provides compelling evidence to further investigate treating these patients with ICI, potentially in combination with targeted therapies, as has been used in *BRAF*-mutant melanoma^57^—with the important caveat that further functional and pre-clinical studies are necessary before considering a trial in human patients. Prior studies have reported that elevated MAPK pathway activity is predictive of responses to ICI in adult glioblastomas, possibly relating to the influence of the MAPK pathway on myeloid cell populations^34,58^. Given that the *BRAF* mutation strongly drives MAPK pathway activity, there may be synergistic benefits between genomic drivers and high levels of inflammation when using immunotherapy to treat this group of patients that is at high risk of progression from their tumor.

Our analysis of MMRD HGGs treated with ICI supports using TIS as a biomarker in these patients for predicting immunotherapy treatment response in conjunction with TMB. These two metrics provide complementary information, as TIS is a measure of immune activation, while TMB is an indicator of neo-antigenicity. The best outcomes were observed in patients with both elevated TIS and TMB, including several patients with recurrent high-grade gliomas who had progression free survival of 3 years or longer with ICI treatment. Conversely, patients with low TIS levels had worse outcomes, even for those with very elevated TMB. An important clinical implication of this finding is that patients with both high TMB and high TIS may be candidates for radiation-free and chemotherapy-free treatment, given their excellent response to immunotherapy^59^.

In summary, characterization of the TIME across pediatric brain tumors provides potential prognostic clues and suggests treatment strategies for further investigation. While evidence to date has not shown success of current immunotherapeutic strategies in most adult glioma patients^13,60^, our findings indicate profound differences between the immune response in pediatric brain tumors compared to adult tumors. Our prior work has demonstrated unprecedented results in treating germline MMRD brain tumors with ICI^7,8^. The current study strongly suggests that other subgroups of patients may benefit from this treatment.

This study illustrates that characterization of the TIME is a critical piece of information alongside driver mutation and TMB for predicting response to immunotherapy, and should be routinely evaluated in the diagnostic workup of pediatric brain tumors and likely many other cancers. While we have largely presented our results in the context of histologically and genetically defined tumor subgroups, there is substantial variation within each of these groups. This underscores that despite the clear relationships between TIME and tumor histology/genetics, knowledge of these is not sufficient to predict the inflammatory response, and it is necessary to evaluate the TIME in each individual tumor, including at multiple time points if available. With numerous novel immunotherapeutic agents in clinical trials, there will be an increasing ability to customize the treatment strategy to the individual patient, which requires accurate characterization of the immune landscape in each tumor^61^.

The diagnostic framework used by pathologists for CNS tumors has undergone a paradigm shift over the past decade, with tumors now reported using an integrated-framework that reports both histology and genomics^62^. In other cancers, such as colorectal cancer, the TIME has prognostic significance that can exceed the standard pathologic risk stratification methods (stage and grade)^1,2^. We envision that in the next decade this will expand to other cancers including CNS tumors, in which the tumor-immune microenvironment is established as another diagnostic “axis” that forms an essential component of personalized medicine.

## Methods

### Study approval and design

This study was approved by the Research Ethics Board (1000055059) at the Hospital for Sick Children (SickKids), and complied with all relevant ethical regulations and Declaration of Helsinki principles. Informed consent from legal guardians was obtained for everyone accrued after 2009, and patient samples from before 2009 were used following REB-approved waiver of consent. Participants were not compensated as no additional costs were incurred for their participation. Sex and gender were not considered in the study design, as the focus of this study was on the TIME as it relates to tumor type, histology, location, and genetics.

### Tumor material and patient characteristics

Study materials profiled at SickKids (referred to as “SickKids cohort”) included frozen or formalin-fixed paraffin-embedded (FFPE) tissue from 571 patient samples from Toronto (SickKids and other hospitals; 491 samples from 475 patients) and the International Replication Repair Deficiency Consortium (IRRDC; 80 samples from 59 patients). The specific source for each individual patient is provided in Supplementary Data 3 (“SickKids cohort info”) under the “Source” column. Genetic characterization of pediatric LGG^53^, DMG, and hemispheric HGG^63–65^ has previously been published (see data availability below). For the adult and young adolescent (AYA) patient cohort, *IDH* mutational status was extracted from our clinical database (manuscript under review) based on the clinical testing results. Clinical data for LGG (all Toronto patients) are provided in Supplementary Data 9, for non-MMRD HGG/DMG (all Toronto patients) in Supplementary Data 13, and for MMRD HGG (mainly IRRDC patients) in Supplementary Data 17.

Sample types were categorized based on a combination of histology, genomic, and clinical features (patient age and location). The pediatric-type HGGs were classified as DMGs for tumors that were located in the brainstem or thalamus/basal ganglia and PHGG for hemispheric tumors (Supplementary Data 13). The adult and young adolescent (AYA) patient cohort consisted of *IDH*-mutant and *IDH*-wildtype diffuse gliomas from patients aged 15–40. *IDH*-mutant diffuse astrocytomas (WHO grade 2–3, *n* = 25) and oligodendrogliomas (grade 2, *n* = 10) were considered LGG. *IDH*-wildtype glioblastomas (grade 4, *n* = 16), *IDH*-mutant astrocytomas (grade 4, *n* = 17), and *IDH*-mutant anaplastic oligodendrogliomas (grade 3, *n* = 5) were classified as HGG. The distinction between PHGG and AYA HGG was based predominantly on age, with patients who were below 15 years at diagnosis considered PHGG.

The pediatric-type LGGs included all low grade-gliomas/glioneuronal tumors in patients <age 18 at diagnosis, except for a small number of samples with an *IDH* mutation (Supplementary Data 9). The most common molecular alterations in the pediatric LGG were *BRAF* fusions (*n* = 79; 77 with *KIAA1549-BRAF*, 1 each *CLCN6-BRAF*, *TAX1BP1-BRAF*) and *BRAF* SNVs (*n* = 38; 35 with BRAF V600E, 1 each with V600ins, T599dup, and D594N), The most common histologic diagnoses were pilocytic astrocytoma (*n* = 83), ganglioglioma (*n* = 29), and diffuse astrocytoma/low-grade astrocytoma NOS (*n* = 26), while many rare diagnoses were represented in lesser numbers. Importantly, the *BRAF*-fused tumors were selected to enrich for recurrent and otherwise atypical cases and therefore are not representative of the population-level survival outcomes that we have previously published^53^.

The MMRD HGG tumor cohort included 83 samples from 62 patients (3 samples from Toronto, 80 from IRRDC). For survival analysis, we used 40 patients from the IRRDC who were treated with immune checkpoint inhibitors, and had (1) pre-treatment tissue available for NanoString profiling, (2) tumor mutation burden data, and (3) sufficient follow-up on treatment for analysis. Several of these patients had multiple resections prior to ICI treatment initiation, and for these the most recent pre-treatment tissue (i.e. closest to the time that treatment began) was used for analysis. See below (“International replication repair consortium clinical trials” section) for more detail regarding the clinical management of these patients, including clinical trial enrollment.

Outside of the MMRD cohort, the patients included in this study were treated with standard of care according to best practices/guidelines at the time of treatment—although recognizing that this study includes samples from a twenty-year period and treatment protocols have shifted over time. The majority of samples were pre-treatment, except where multiple longitudinal samples existed for the same patient (see Fig S3d; samples denoted as T1, T2, etc in the Study ID in Supplementary Data). Some sample types, particularly post-mortem samples of diffuse midline gliomas, have a greater proportion of post-treatment samples, however it was not found that treatment impacts the variables studied in this paper (see Fig S7d, Supplementary Data 14). Importantly, all survival-based analysis (for LGG in Figs. 3, 4 and MMRD HGG in Fig. 6) was performed only using pre-treatment samples.

Given the fact the gene expression testing, regardless of the platform, provides values that can only be interpreted in relation to a reference dataset, it was critical to select a reliable control dataset—however this was challenging given that truly normal brain tissue is never surgically resected. While tissue from epilepsy resections is not entirely normal due to the effects of refractory seizures, this was the best reference set that could be obtained, and all slides were reviewed by two neuropathologists (CH, AL) to exclude tissue with inflammation. The alternative would have been to use post-mortem tissue, however most cases that have the brain examined at our site have some degree of abnormal neuropathologic findings and hence are not suitable for this use.

### International replication repair consortium (IRRDC) clinical trials

The International Replication Repair Deficiency Consortium (IRRDC) is based at SickKids, Toronto. The IRRDC has enrolled >200 patients with confirmed /suspected replication repair deficiency from 45 countries since 2007 (see ref. ^8^ for more detail). This study included 59 IRRDC patients with 80 RRD tumors, among whom 1 was treated with chemo-radiation alone, and the remaining 58 received additional therapy with immune checkpoint inhibitors (ICI) following progression on their standard-of-care treatment (Supplementary Data 17, 18). Among patients treated using ICI, 4 were treated on a recently published international, prospective clinical trial (NCT02992964; https://clinicaltrials.gov/study/NCT02992964)^66^. Their clinical outcomes were reported as per the trial’s objectives (radiological objective response, PFS, adverse events). The current manuscript includes longer follow-up and OS data since they came off-study, which have never been published before as well as the TIME and TIS data. Important, analyses performed here represent non-prespecified exploratory outcomes for NCT02992964.

The remainder were treated on a global registry study conducted by the International RRD Consortium, either off-label or through compassionate drug access, as per NCT02992964 clinical trial protocol guidelines (including for monitoring safety and stopping rules for toxicity) and as previously reported by our group^8,67^. Specifically, ICI treatment for patients with RRD-HGG involved treatment using anti-PD1, either nivolumab 3 mg/kg q2-weeks, (maximum = 240 mg/dose) in the majority, and rest using pembrolizumab 2 mg/kg q3-week (maximum=200 mg/dose), dependent on local logistics and physician choice. For patients receiving non-standard of care therapy, drugs were obtained either off-label or through compassionate drug access, as described in refs. ^8,67^. Monthly meetings (and additional ones as needed) were coordinated to track progress, address any safety concerns, and collect data in real-time. The immune TME and TIS data and correlation with OS are being presented for the first time for the entire cohort, including for patients whose radiological response and PFS were previously reported.

### NanoString gene expression profiling

Gene expression profiling was carried out using the NanoString nCounter system^68^, with a customized CodeSet composed of a total of 103 genes (see Supplementary Data 4 for panel details and Supplementary Data 1 for normalized counts matrix). A major advantage of the NanoString platform (compared to RNA sequencing, for example) is its robust results on formalin-fixed paraffin-embedded tissue (FFPE), which allowed us to use archival tissue samples up to 20 years old. Total RNA was extracted from FFPE and snap-frozen tissue using RNAstorm FFPE and fresh frozen kits, respectively, as per the manufacturer’s instructions (CellData). RNA concentration was measured (Nanodrop) and RNA integrity assessed using the Agilent 2100 bioanalyzer. Using a minimum of 100 ng of extracted RNA, gene quantification was performed on the NanoString nCounter platform per the manufacturer protocols. During assay development, intra-run (3 replicates in the same run) and inter-run (3 replicates in different runs) reproducibility was evaluated using 4 samples. All attempts at replication were successful.

### NanoString quality control, data processing, and analysis

All data analysis used Bioconductor version 3.14 or 3.16 packages in R version 4.1 or 4.2, respectively, unless otherwise stated. Raw NanoString data (RCC files) was processed using the NanoStringQCPro package (v1.26.0). For quality control (QC), the geometric mean of the five housekeeping (HK) genes (DDX50, EIF2B4, MRPS5, SAP130, TLK2) was used as a metric of overall RNA quality and quantity, and samples with a value of <100 were excluded. Out of a total of 688 samples (age range 0-32 years) that were run during test development and data generation, 52 (7.5%) failed QC and were excluded from further analysis (see Fig S14a, Supplementary Data 21). There was a strong inverse correlation in years between sample age and HK gene levels (Fig S14b; *R* = −0.68) and accordingly the age of QC fails (median = 14.4 years) was substantially higher than QC passes (median = 6.6 years, *p* = 2.3 × 10^−10^; Fig S14c). That said, sample quality cannot be predicted solely by sample age; there were samples up to 20 years of age with excellent quality and resulting NanoString data, while samples <5 years old occasionally failed QC. NanoString data was validated in comparison to IHC for selected genes (see “Immunohistochemistry staining and scoring” below), as well as matched RNA sequencing for 15 samples (Fig S14d, Supplementary Data 22).

The data from all samples was normalized together using housekeeping genes and positive control genes as recommended in the NanoString data analysis guidelines. Briefly, for each sample a normalization was performed sequentially using first the positive controls and then the housekeeping genes. For each sample the geometric mean of the positive controls was calculated (geomean_control)_. The geometric mean of all the geometric means was then calculated (geomean_geomeans_) and this was used to calculate a sample specific normalization factor (geomean_geomeans_ /geomean_control_), which was multiplied by the gene counts for each sample. The same steps were then taken using the housekeeping genes instead of the positive controls, to provide the final normalized gene counts.

To provide an overall assessment of the immune microenvironment, we used the tumor inflammation signature (TIS), which is also referred to in literature as “T-cell inflamed gene expression profile”. This is a well-validated 18 gene score that includes probes involved in antigen presenting cell abundance (*PSMB10*, *HLA-DQA1*, *HLA-DRB1*, *CMKLR1*), T cell/NK cell abundance (*HLA-E*, *NKG7*, *CD8A*), interferon activity (*CCL5*, *CXCL9*, *CD27*, *CXCR6*, *IDO1*, *STAT1*), and T-cell exhaustion (*TIGIT*, *LAG3*, PD-L1/*CD274*, PD-L2/*PDCD1LG2*, B7-H3/*CD276*). The individual gene weightings for the TIS score were not disclosed in the original publication^51^; we obtained them, upon correspondence with the authors, from their patient filing WO2016094377 in claim 21c (available at https://patents.google.com/patent/WO2016094377A1). The scores for each sample were calculated as described in the reference papers^29,51^, using matrix multiplication of the log2 transformed gene counts by the corresponding weights for each gene. Histologic assessment of samples with high, medium, and low TIS scores, respectively demonstrated morphologic correspondence to the presence of inflammatory infiltrates in the tissue sections (Fig S2b), while TIS expands upon morphologic assessment by incorporating markers of antigen presenting cells (APC) and T-cell exhaustion.

Heatmaps were created using ComplexHeatmap (v2.10.1)^69^. Unsupervised hierarchical clustering was performed using ConsensusClusterPlus (v1.58.0) with the Pearson correlation as the distance^70^. A wide range of cluster numbers was evaluated (2-10), which were evaluated using consensus matrices, consensus cumulative distribution function plots, delta area plots, tracking plots, and item-consensus plots. Differential expression analysis was performed using row-wise t-tests through the genefilter (v1.76.0) package, with the *p*-values corrected using the Benjamini & Hochberg method.

### Immunohistochemistry staining and scoring

IHC was performed in the CLIA approved pathology laboratory at the Hospital for Sick Children. Four-micron thick sections of formalin-fixed paraffin-embedded (FFPE) surgical specimens were stained using the Dako Omnis automated stainer with the EnVision Flex detection kit. The following primary antibodies were used: PD-L1 (clone:28-8, Rabbit monoclonal Abcam, 1:500 cat no:ab205921), CD68 (clone:PG-M1, Dako-Omnis, ready to use, cat no:GA613), CD8 (clone:c8/144B, Dako-Omnis, ready to use, cat no:GA623), CD3 (polyclonal rabbit, Dako-Omnis, ready to use, cat no:GA503).

Slides were scanned at minimum 200x magnification using an Aperio AT2 scanner. Subsequent visualization and analysis of whole slide images (WSIs) was done with QuPath (version 4.3.0). For comparison of IHC to NanoString gene counts, matched tissue sections from the same blocks were used. All assessment of the results of immunohistochemical staining and tumor content was performed by a board-certified neuropathologist (AL), who was blinded to NanoString data at the time of assessment (Supplementary Data 2). CD3and CD68 were scored using the QuPath “Positive cell detection” function. Regions of interest were selected by a neuropathologist to include the maximum possible tissue area, while excluding areas of artifact and non-interpretable tissue. Slides were scored as the percentage of immunohistochemically positive cells among all detected cells for the full slide area. Given the well-recognized challenges in assessing PD-L1 IHC^71^ it was not feasible to quantify digitally. Instead, PD-L1 was assessed semi-quantitatively over the entire tissue area on the slide, excluding areas of artifact and otherwise poor-quality histology. Tumors were categorized as having high (>5% PD-L1 positive tumor and immune cells), low (≥1% to <5% PD-L1-positive tumor cells and immune infiltrates) or no staining (<1% PD-L1 positive tumor and immune cells).

### Whole exome sequencing (WES)

MMRD tumors had matched tumor and blood whole exome sequencing (WES) performed at the Centre for Applied Genomics (TCAG) at SickKids and aligned to hg38, as previously reported by our group^8^. Somatic variants were called using Mutect2^72^, filtered for quality, a minimum 0.05 VAF cutoff, and common SNPs (dbSNP b151^73^), and annotated with Funcotator (both from GATK v4.2.3.0^74^). TMB was calculated by counting a total number of somatic single nucleotide variants divided by a total number of callable bases in megabases (~50 Mb). To filter for impactful variants, we then selected those with impacts on coding sequences (missense, truncating, frameshift, etc.) or at splice site junctions. Variants which had benign or likely-benign annotations in ClinVar (2018-04-29)^75^ were excluded, as well as variants without an annotation in the COSMIC Cancer Gene Census^76^ (v97; Funcotator Data Source v1.7.20200521g). (See Supplementary Data 18, 19 for summaries of variants).

### NGS panel sequencing

DNA was extracted from FFPE blocks using DNAstorm (CellData). Libraries were generated using custom hybrid capture probes (Twist Bioscience) targeting commonly altered glioma driver genes (LN, CH, in preparation) and sequenced on NextSeq instruments (Illumina). Samples were aligned to genome build hg19 and analyzed using the DRAGEN pipeline with default settings in BaseSpace (Illumina) to identify small nucleotide variants (SNVs), insertions and deletions. Fusions were identified using Illumina DRAGEN and Arriba (v2.4.0)^77^.

### Pediatric Brain Tumor Atlas (PBTA) data analysis

810 samples from the PBTA^23^ were analyzed (see Supplementary Data 5 and 14). Clinical annotations were downloaded from PedcBioPortal (https://pedcbioportal.kidsfirstdrc.org/study/clinicalData?id=openpbta) and tumors were grouped to best correspond to the SickKids tumor types based on the “cancer type” and “cancer type detailed” columns. The cancer types “Low-grade astrocytic tumor” and “Neuronal and mixed neuronal-glial tumor” were grouped as LGG, “Diffuse astrocytic and oligodendroglial tumor” as HGG, and “Pre-cancerous lesion” as non-tumor control tissue. For HGGs, the subtypes “Brainstem glioma- Diffuse intrinsic pontine glioma” and “Diffuse midline glioma, H3 K28-mutant” were grouped as DMG, while PHGG included “High-grade glioma/astrocytoma (WHO grade III/IV)”, “High-grade glioma/astrocytoma, H3 wildtype”, and “High-grade glioma/astrocytoma, H3 G35-mutant”. Ependymomas were grouped as “RELA-fused” and “non-RELA” for all other types. Otherwise, we used the PBTA provided labels for specific cancer types.

Harmonized gene expression data was downloaded from Cavatica. The RSEM gene-level quantification data (rsem.genes.results.gz files) were loaded with the Bioconductor tximport (v1.22.0) package. To enable comparison between samples with poly-A and total RNA-seq libraries, the total RNA-seq samples were filtered only for protein-coding genes and rescaled to TPMs. The TPM normalized counts (from the RSEM abundance column) were multiplied by 10 to put them in a more similar range to the NanoString gene counts, and then log2 transformed. TIS calculation and other downstream analyses were then performed as previously described for the NanoString data. Importantly, given the difference in platform and normalization between the NanoString data and PBTA data, the absolute values of TIS scores are not directly comparable with each other, and instead are interpreted in relation to each dataset’s respective non-tumor brain reference samples. Pathway scores were calculated using the Bioconductor progeny package (v1.20.0)^33^. Immunologic signature gene sets from Bagaev et al^5^. were calculated using gene set variation analysis with the Bioconductor GSVA package (v1.46.0)^78^.

For DNA sequencing data analysis, processed data from release v22-20220505 was downloaded from Cavatica (https://cavatica.sbgenomics.com/u/cavatica/openpbta). Consensus SNV (pbta-snv-consensus-mutation.maf.tsv.gz) and CNV (consensus_seg_annotated_cn.tsv) calls were used for correlation of genomic features with inflammation levels in high-grade gliomas, with 4 ultra-hypermutant samples removed to avoid skewing the results. SNVs were filtered for protein-altering variants and categorized as missense, frameshift indels, nonsense, or splice region. For quantification of total numbers of copy number alterations, adjacent segments with the same copy number were merged, and alterations were categorized as gains, amplifications (5 or more copies in a diploid sample), losses, or deep deletions (0 copies in a diploid sample). For correlation of TIS with COSMIC mutational signatures, we used the signatures calculated with deconstructSigs (cosmic_signatures_results.tsv) through the Open-PBTA (https://github.com/AlexsLemonade/OpenPBTA-analysis) workflow.

### Statistics

Pairwise comparisons of continuous variables (gene expression counts and the TIS) was performed with the R ggpubr (v0.4.0) package using two-tailed T-tests with Holm adjustment reported on the boxplots, unless otherwise stated in figure legends. Αll boxplots show the median and interquartile range (IQR) of the underlying data with whiskers extending to ±1.5 IQR from boxes. Correlation values for correlation matrices with scaled values were calculated using the Pearson correlation and for scatter plots with unscaled values using the Spearman coefficient (to decrease the impact of outliers). Other statistical tests were performed as indicated in the text.

Survival analysis was performed with the R survival (v3.4-0), survminer (v0.4.9), and survivalAnalysis (v0.3.0) packages, using Kaplan Meier curves, the log-rank test for univariate analysis, and Cox proportional hazards test for multivariate analysis. For PLGG, the endpoint used was progression-free survival, defined as the time from initial surgery to first clinical or radiographic progression, with patients who did not progress censored at the latest follow-up time. The optimal cutoff for dichotomizing groups as high/low TIS were determined using the surv_cutoff function in the R survminer package and *p*-values were calculated through bootstrapping with 1000 iterations, each sampling 90% of the dataset. For multivariate survival analysis of the three BRAF V600E tumor clusters, extent of resection could not be included in the Cox regression due to an absence of progression events in patients with gross total resections; instead patients with incomplete resection only were analyzed as a separate subgroup.

For MMRD samples treated with immunotherapy the endpoint used was overall survival, defined as the time of initiation of immunotherapy to date of death from any cause, with patients censored who were still alive at the most recent follow-up date. Survival analysis separating into 3 groups by quartile (high > 75th percentile, medium 25–75th percentile, low <25th percentile) indicated that only the top quartile had improved response to ICI, therefore the 75th percentile cutoff TIS = 9.13 was rounded to 9 for dichotomizing samples as high/low TIS.

### Reporting summary

Further information on research design is available in the Nature Portfolio Reporting Summary linked to this article.

### Supplementary information

Supplementary Information

Peer Review File

Description of Additional Supplementary Files

Supplementary Data 1-22

Reporting Summary

## Data Availability

The NanoString data newly generated on our cohort is available at GEO under GSE227756. Newly generated panel sequencing data of high-grade gliomas is available at EGA under study EGAS50000000221 and dataset EGAD50000000326. The data are available under controlled access to comply with data protection regulations, and can be accessed by application to the data access committee via C.H. (cynthia.hawkins@sickkids.ca). The remaining data, including normalized NanoString gene counts (Supplementary Data 1), are available within the Supplementary Data file with the paper; all figures can be reproduced from the Supplementary Data. Previously published WES data from tumors with matched germline blood for MMRD patients treated with immune checkpoint inhibition^8^ is available on EGA (EGAD00001008036).This can accessed by application to the data access committee via U.T. (uri.tabori@sickkids.ca). Previously published RNA and targeted DNA sequencing data for PLGG^53^ is available on EGA (EGAD00001005987), and can be accessed by application to the data access committee via C.H. (cynthia.hawkins@sickkids.ca). Additional clinical data and molecular characterization (using IHC, FISH, NanoString gene fusion panels, SNP array) are available as source data at the manuscript website 10.1016/j.ccell.2020.03.011. Previously published sequencing data for DMG and hemispheric PHGG^63–65^ is available on EGA for WGS (EGAD00001000814, EGAD00001003305), WES (EGAD00001006450, EGAD00001008279, EGAD00001003305), and RNA-seq (EGAD00001006450, EGAD00001008278). These can be can be accessed by application to the data access committee via C.H. (cynthia.hawkins@sickkids.ca). The publicly available PBTA raw data is available through KidsFirstPortal (https://portal.kidsfirstdrc.org/login) accession codes PBTA-CBTN and PBTA-PNOC. and Cavatica (https://cavatica.sbgenomics.com/u/cavatica/openpbta) upon request to CBTN, and processed summary files are accessible at https://github.com/AlexsLemonade/OpenPBTA-analysis.

## Code availability

Example scripts for processing NanoString data, including normalization and TIS score calculation, are available on GitHub at: https://github.com/adrianblevine/Immuno-oncology_nanostring. DOI: 10.5281/zenodo.10739152.
