# Supplementary material for: Immuno-oncologic profiling of pediatric brain tumors reveals major clinical significance of the tumor immune microenvironment
Source: Nat Commun. 2024 Jul 10;15:5790. doi: 10.1038/s41467-024-49595-1 (PMC11237052; doi:10.1038/s41467-024-49595-1)
Supplement: Supplementary file 1 — Supplementary Information [file 41467_2024_49595_MOESM1_ESM.pdf]

**SUPPLEMENTARY INFORMATION:**

Figure S1: Additional NanoString-IHC comparisons.

Figure S2: Overview of inflammation levels in pediatric brain tumors.

Figure S3: TIS scores for ependymoma and embryonal tumor subtypes.

Figure S4: TIS correlations with pathway activation scores and mutational signatures for PBTA samples.

Figure S5: Pediatric LGG TIS levels by genetic driver alteration.

Figure S6: Additional analysis of BRAF V600E subgroups.

Figure S7: Additional figures for high-grade gliomas.

Figure S8: Representative immunohistochemistry for diffuse midline gliomas.

Figure S9: TIS correlations with genomic features for pediatric HGG.

Figure S10: TIS correlations with genomic features for MMRD HGG.

Figure S11: Additional survival analysis for MMRD HGG.

Figure S12: Gene expression levels for MMRD HGG.

Figure S13: Additional pre/post-ICI IHC for MMRD HGG.

Figure S14: NanoString assay validation and QC data.

**Figure S1** (related to Fig 1)

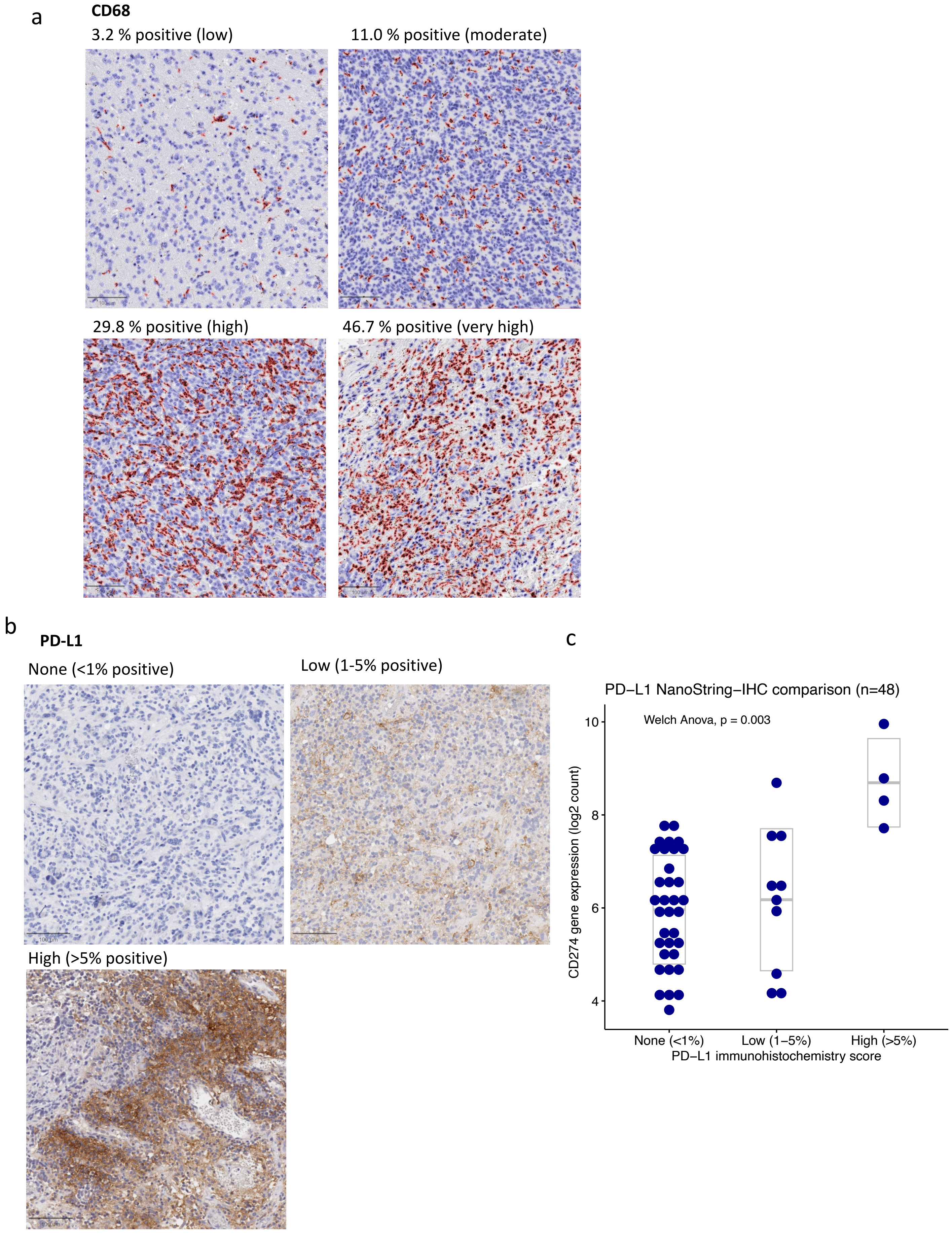

**Figure S1: Additional NanoString-IHC comparisons.**

(a) Example images of digital quantification of CD68 IHC. Representative areas of whole slide images were selected for visualization, with the percentage of positive cells in the entire slide indicated. Scale bar is 100 µm. Images taken at 100x magnification.

(b) Representative images of semi-quantitative PD-L1 scoring by a neuropathologist as none (<1% positive cells), low (1-5% positive) , or high (>5% positive). Scale bar is 100 µm. Images taken at 100x magnification.

(c) Stripchart of comparison between CD274 gene expression by NanoString and PD-L1 immunohistochemical scoring. Boxplots show the median value and interquartile range. P-value between the three groups by Welch's ANOVA.

**Figure S2** (related to Fig 1)

a

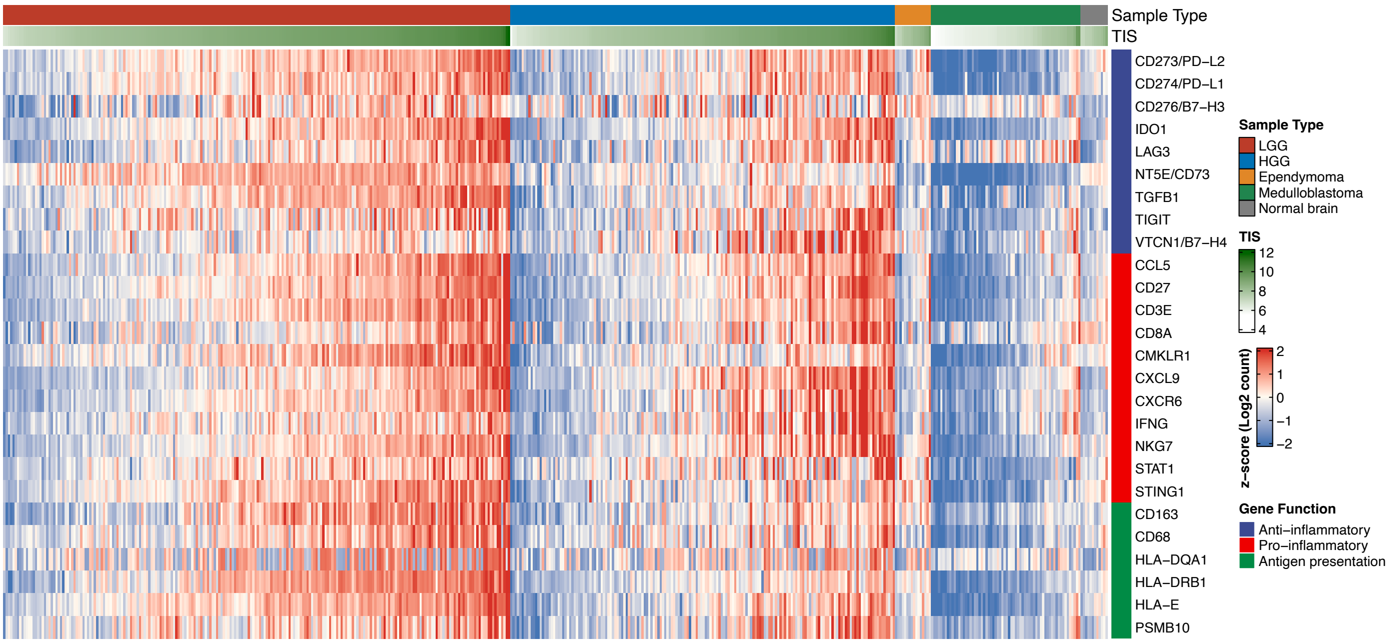


b

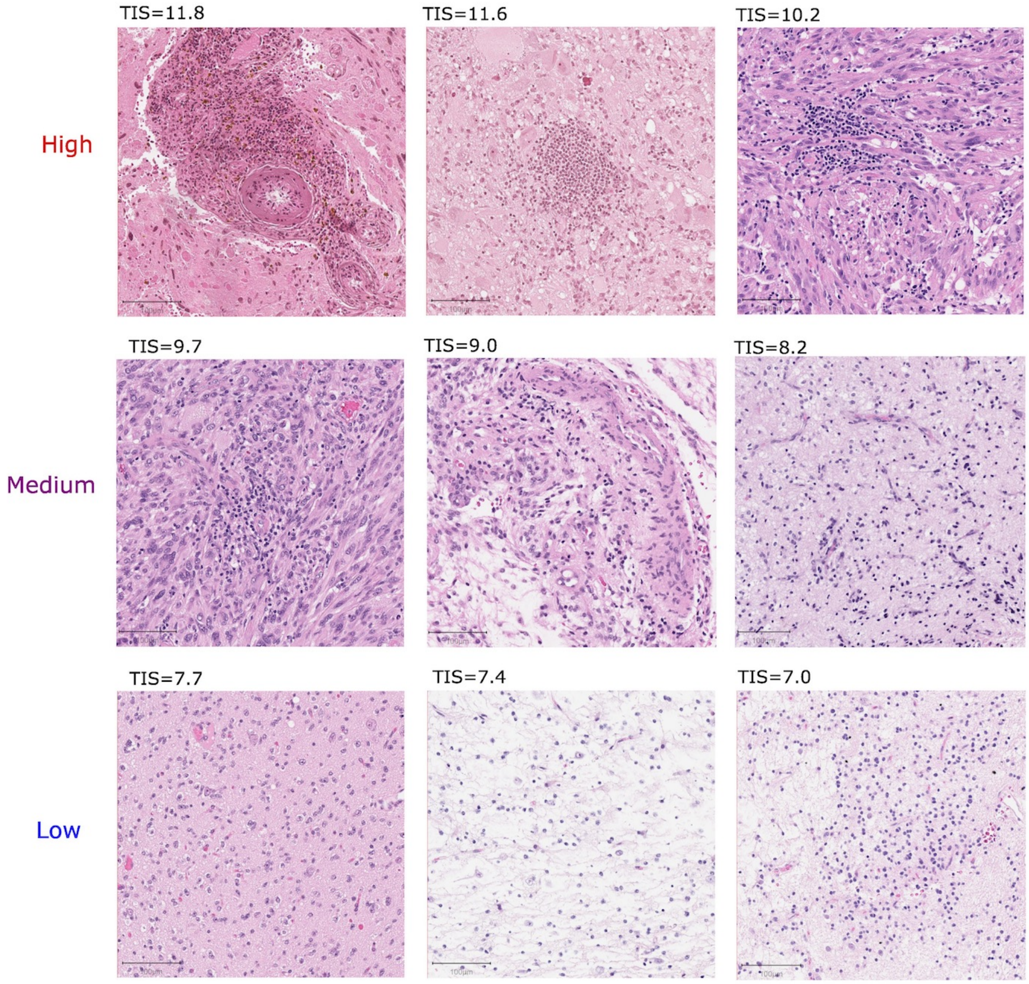

**Figure S2: Overview of inflammation levels in pediatric brain tumors.** (a) Heatmap of gene counts of tumor inflammation signature (TIS) genes and other selected probes across the most common types of PBTs. (b) Representative histologic images of samples with high (>10), medium (8-10), and low (<8) TIS scores. Scale bar is 100 μm. Images taken at 100x magnification.

# **Figure S3** (related to Fig 1)

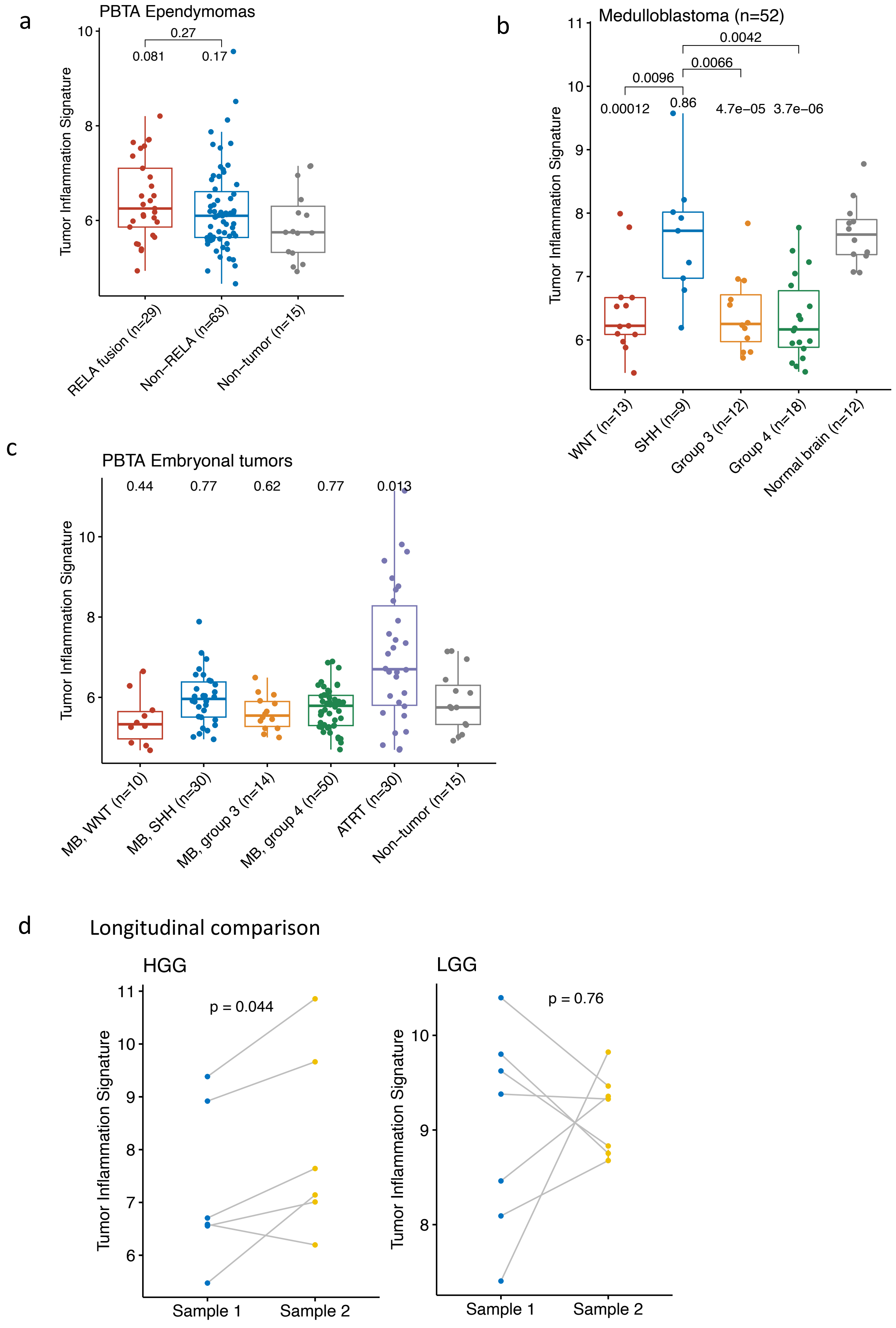

**Figure S3: TIS scores for ependymoma and embryonal tumor subtypes.**

(a-c) Boxplots of TIS scores for (a) PBTA ependymomas, (b) SickKids medulloblastomas, (c) PBTA embryonal tumors. Boxes show the median and interquartile range (IQR) of the data with whiskers extending to ±1.5 IQR. P values by two-tailed T-test with Holm adjustment.

(d) Longitudinal comparison of TIS scores at each timepoint for tumors with multiple samples, excluding those that received immunotherapy. P-values calculated using Wilcoxon signed rank test.

(ATRT: atypical teratoid/rhabdoid tumor; MB: medulloblastoma; ST: supratentorial; PF: posterior fossa; NOS: not otherwise specified)

**Figure S4** (related to Fig 2)

a

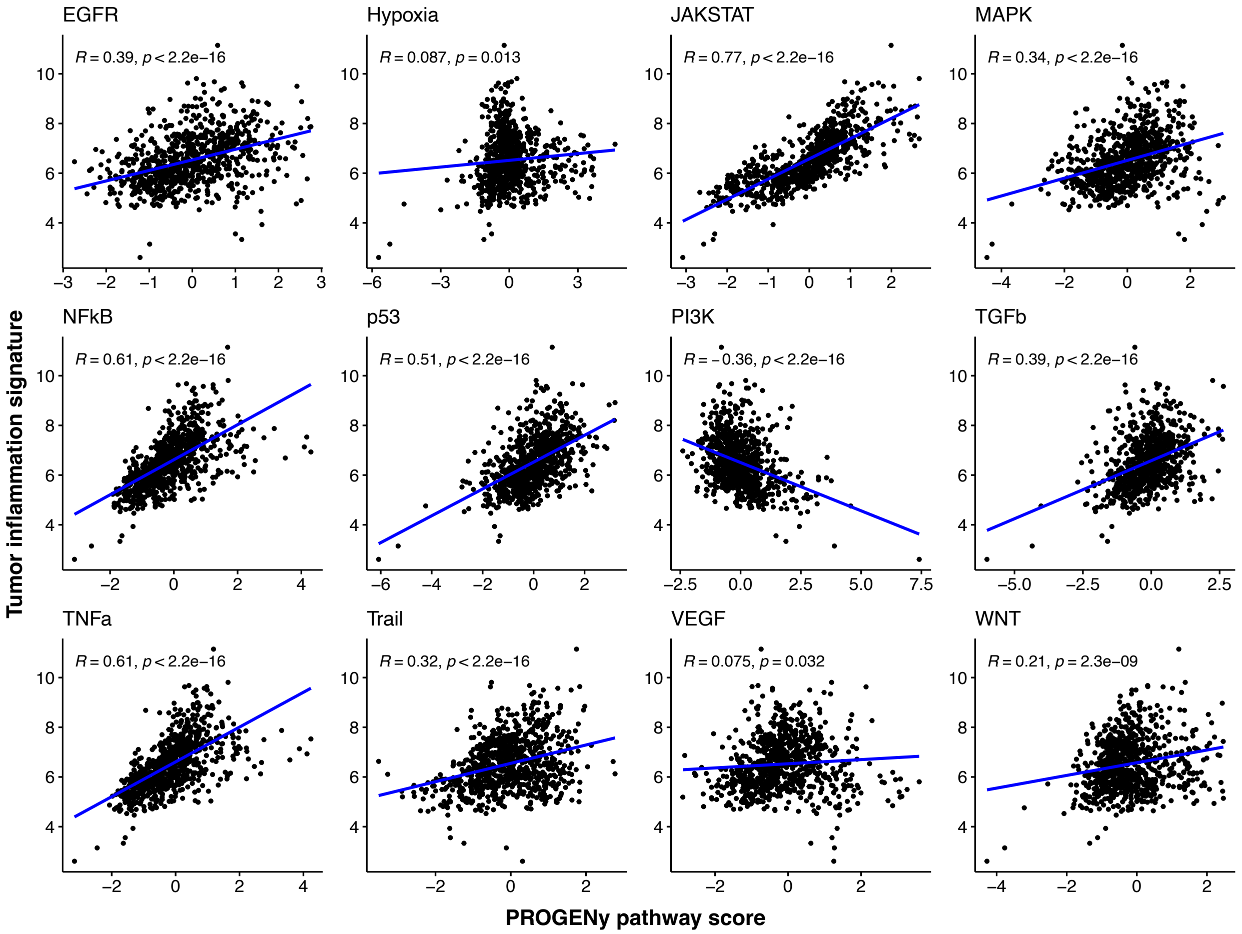


b

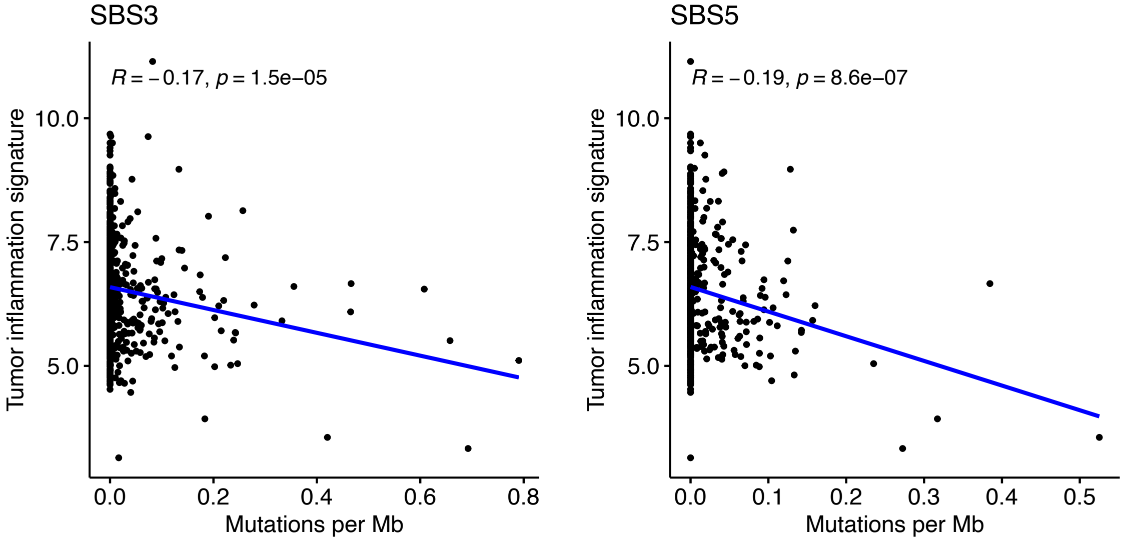

**Figure S4: TIS correlations with pathway activation scores and mutational signatures for PBTA samples.**

(a) Scatter plots of relationship between TIS and PROGENy pathway scores for each individual pathway. (b) Scatter plots of relationships between TIS scores and mutations per Mb attributed to COSMIC SBS3 and SBS5 mutational signatures. N=810 samples from PBTA. R and p-values using Pearson correlation.

**Figure S5** (related to Fig 3)

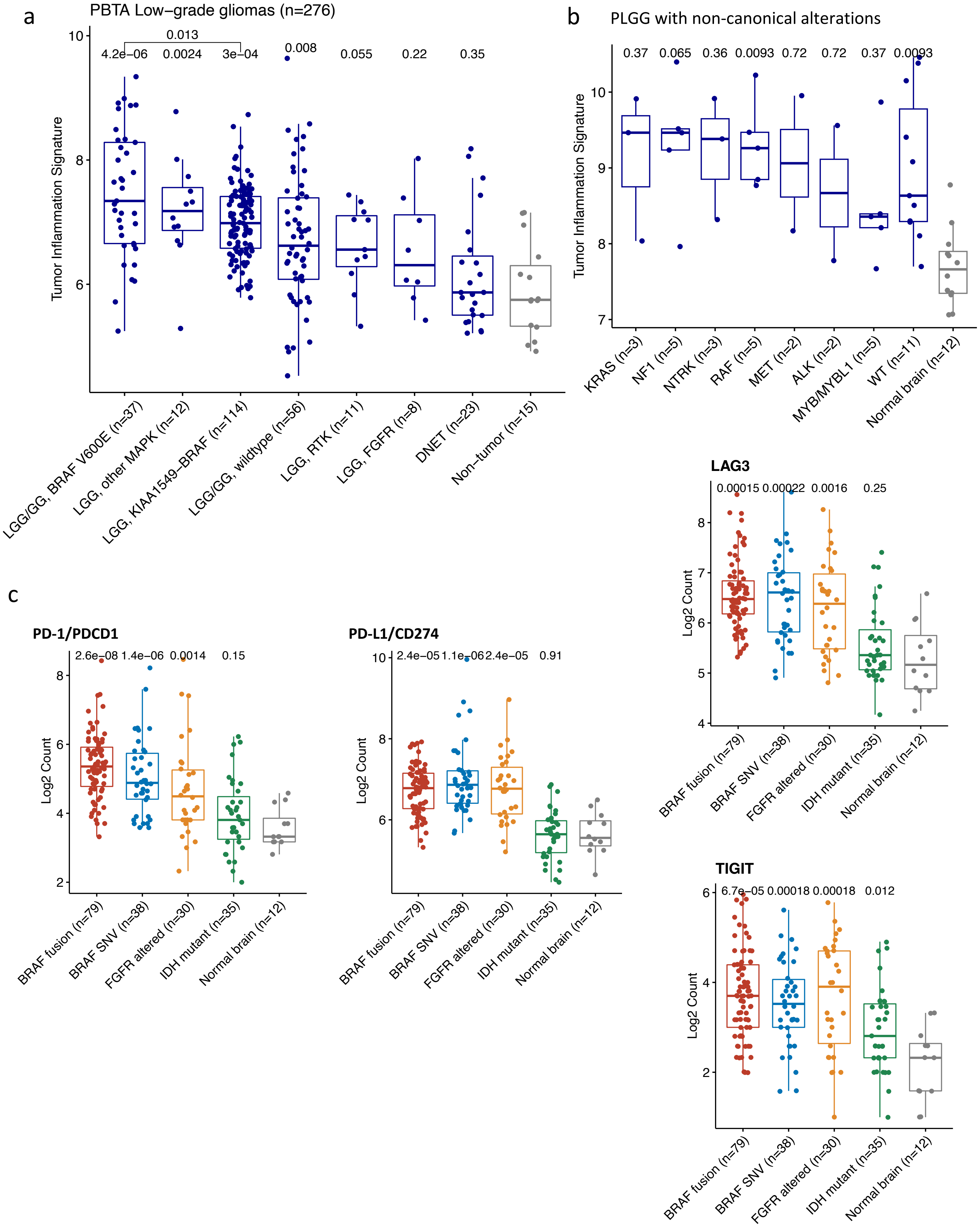

**Figure S5: Pediatric LGG TIS levels by genetic driver alteration.**

(a) Boxplot of TIS scores for PBTA low-grade gliomas and glioneuronal tumors by subtype. Boxes show the median and interquartile range (IQR) of the data with whiskers extending to ±1.5 IQR. P-value by two-tailed T-test with Holm adjustment.

(b) Boxplot of TIS scores for SickKids PLGG samples with rare/non-canonical genomic alterations. P-values by two-tailed T-test with Holm adjustment.

(c) Box plots of PD1, PD-L1, LAG3, and TIGIT gene counts split by LGG subtype. P-values by two-tailed T-test with Holm adjustment. (LGG: low-grade glioma; GG: ganglioglioma; DNT: dysembryoplastic neuroepithelial tumor; RTK: receptor tyrosine kinase; WT: wild-type)

# **Figure S6** (related to Fig 4)

a

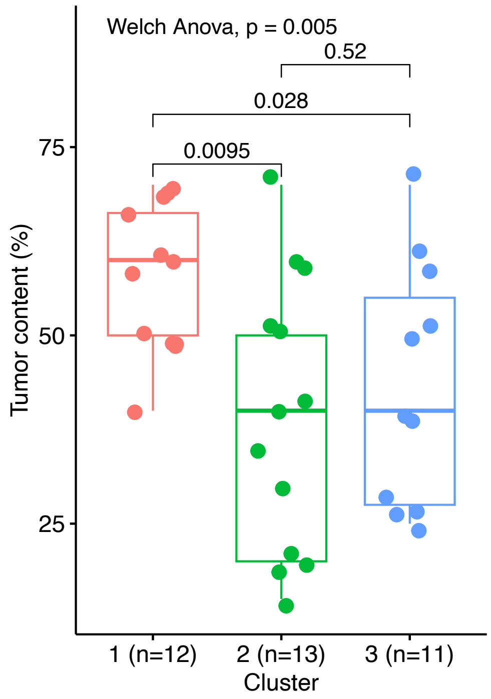


b

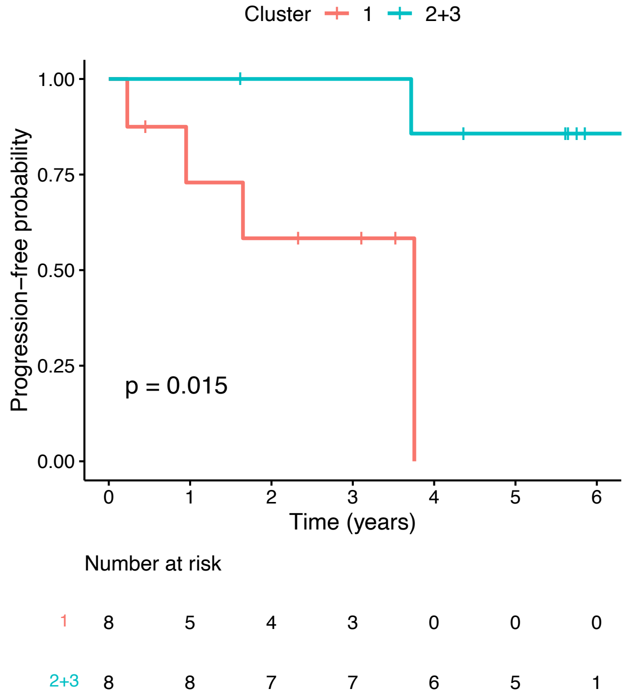


c

| **Univariate survival analysis** | | | | |
|---|---|---|---|---|
| **Variable** | **P (log-rank test)** | **P (Cox)** | **Hazard ratio** | **Confidence interval** |
| Cluster (1 vs 2+3) | 0.005 | 0.025 | 12.42 | 1.36-113.32 |
| Gross total resection | 0.005 | NA | NA | NA |
| CDN2A homozygous deletion | 0.013 | 0.033 | 7.36 | 1.17-46.38 |
| Age (above median) | 0.334 | 0.354 | 0.35 | 0.04-3.21 |
| Location (hemispheric) | 0.489 | 0.496 | 0.54 | 0.09-3.22 |
| Histology (ganglioglioma) | 0.909 | 0.909 | 1.11 | 0.18-6.72 |

d

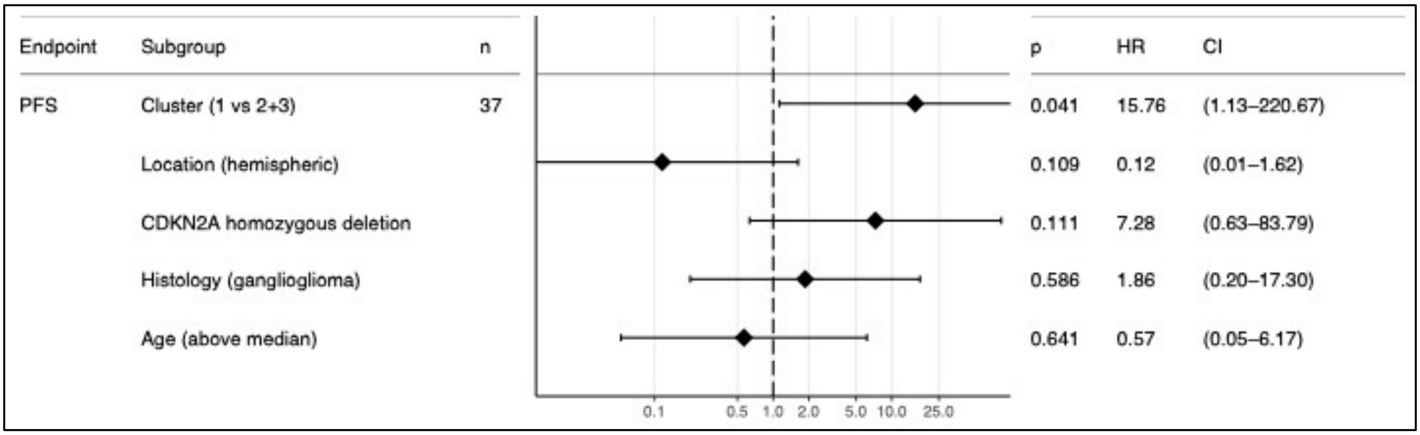

**Figure S6: Additional analysis of BRAF V600E subgroups.**

(a) Boxplot of tumor content assessed by a neuropathologist for the BRAF V600E samples. Boxes show the median and interquartile range (IQR) of the data with whiskers extending to ±1.5 IQR. P-values by two-tailed Wilcoxon for pairwise comparisons and Welch Anova for multigroup comparison.

(b) Kaplan-Meier curves of progression-free survival for LGG BRAF V600E immune cluster 1 compared to cluster 2+3, excluding patients with gross total resections. P-value by log-rank test.

(c) Table of univariate survival analysis for cluster 1 vs cluster 2+3 with P values calculated using both the log-rank test and Cox regression.

(d) Forest plot of multivariate survival analysis for cluster 1 vs cluster 2+3 using Cox regression.

**Figure S7** (related to Fig 5)

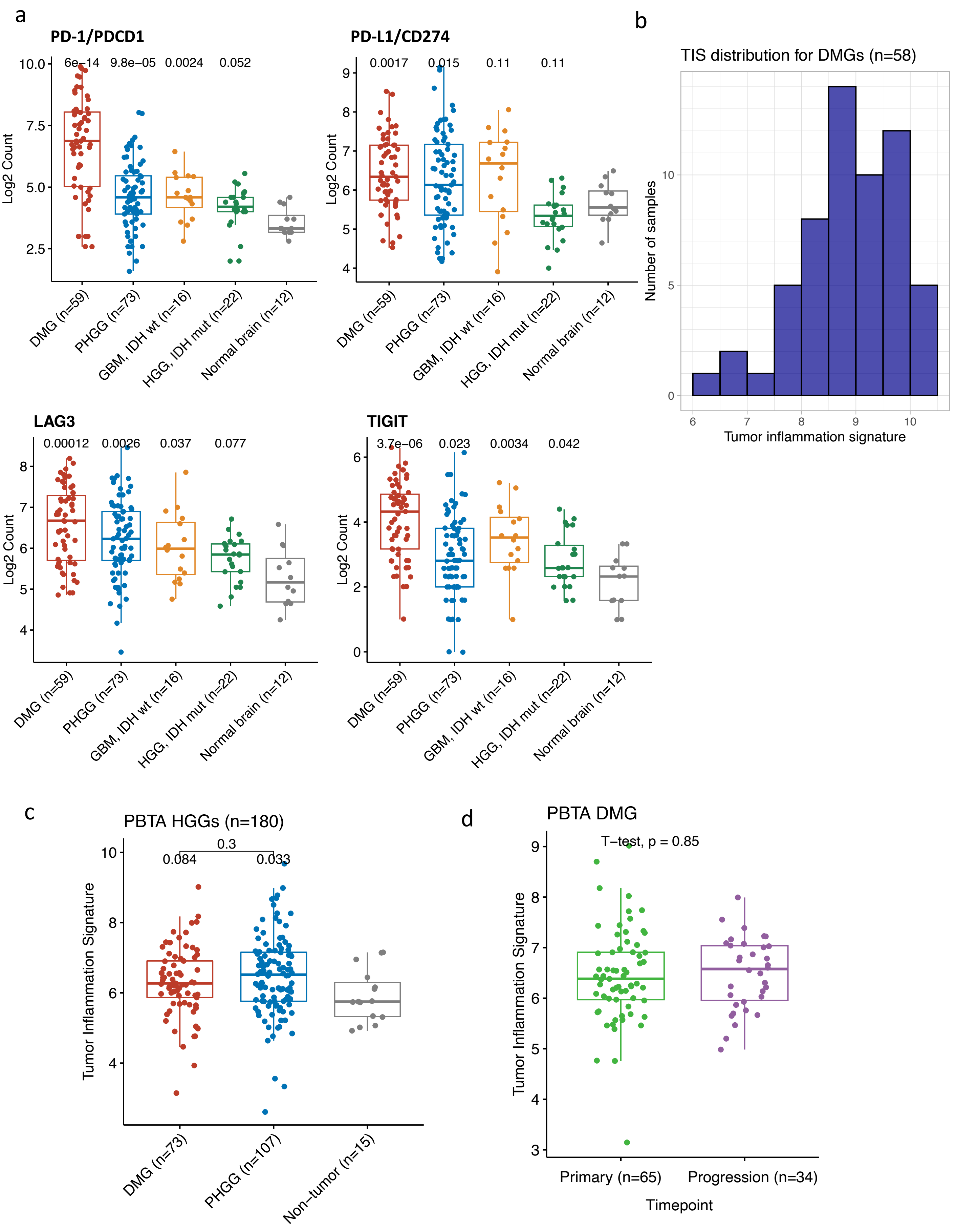

**Figure S7: Additional figures for high-grade gliomas.**

(a) Boxplots of gene counts for PD-1, PD-L1, LAG3, and TIGIT in HGGs. Boxes show the median and interquartile range (IQR) of the data with whiskers extending to ±1.5 IQR. P-values by two-tailed T-test with Holm adjustment.

(b) Histogram of TIS score distribution for diffuse midline gliomas in SickKids cohort.

(c-d) Boxplots of TIS scores for PBTA HGGs (c) comparing midline with hemispheric tumors and (d) comparing primary DMG with progressive/recurrent DMG. P-values by two-tailed T-test with Holm adjustment.

**Figure S8** (related to Fig 5)

a

DIPG126
TIS N/A

DIPG007
TIS=9.31

DIPG005
TIS=8.69

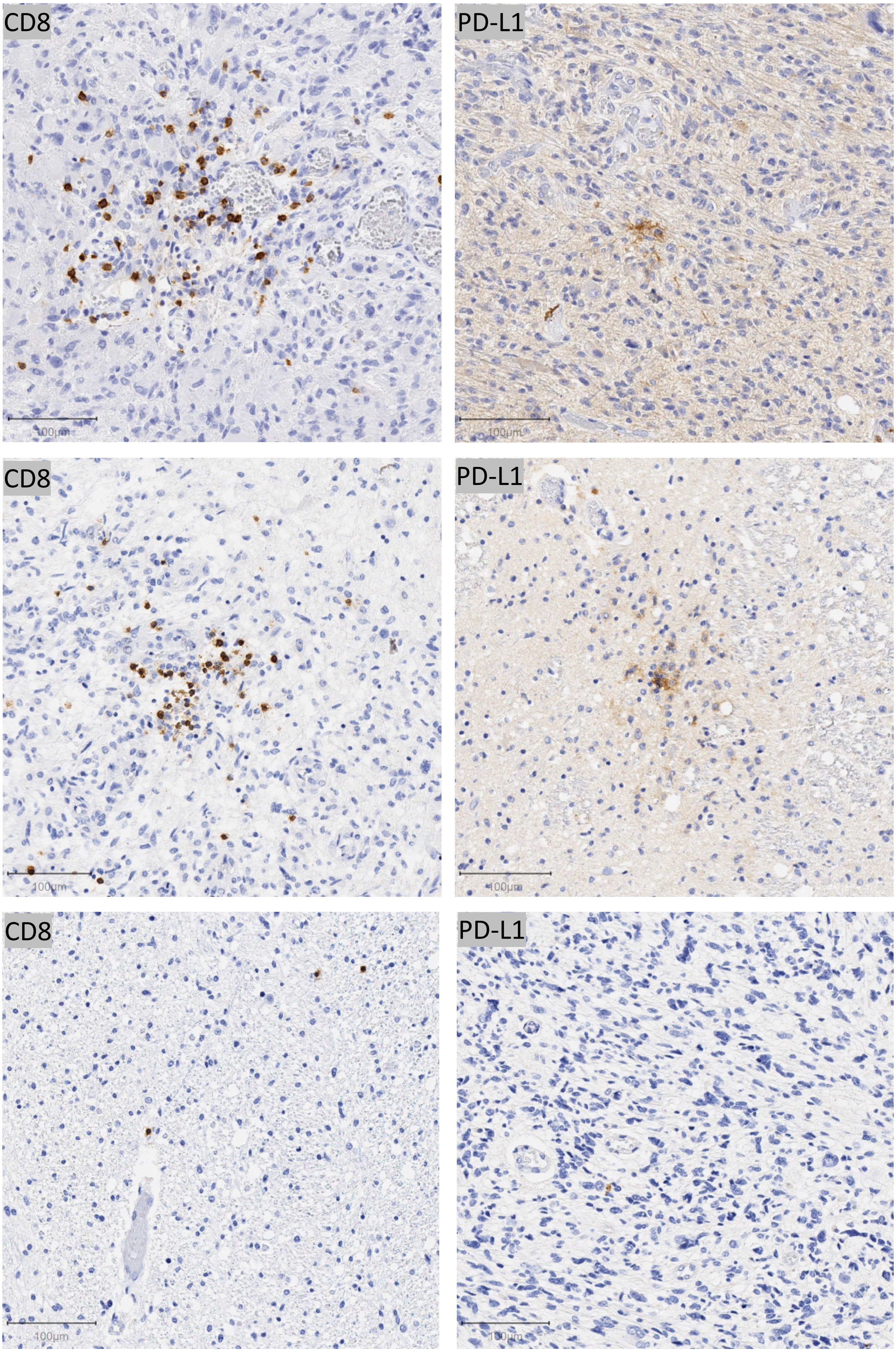

**Figure S8** (continued)

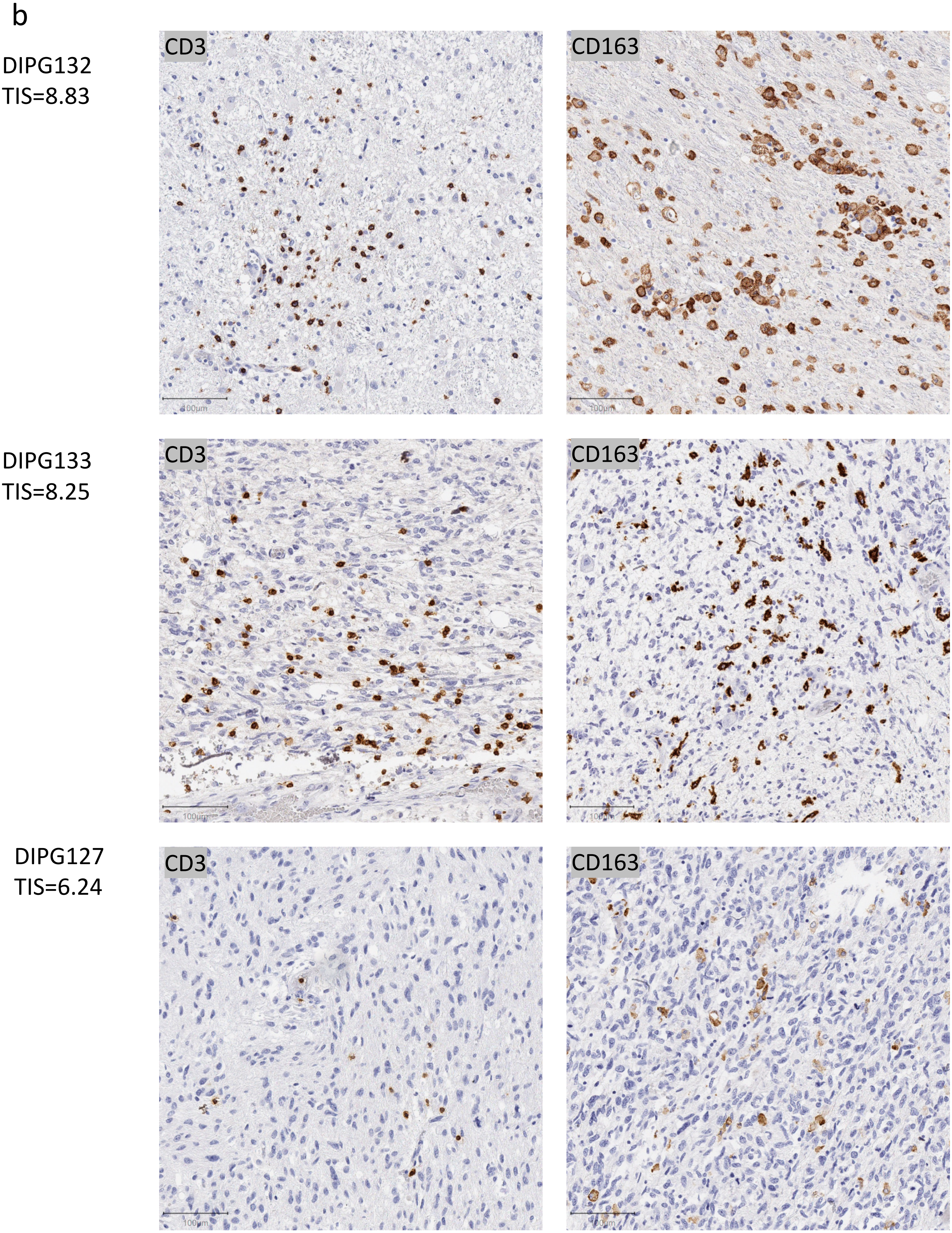

**Figure S8: Representative immunohistochemistry for diffuse midline gliomas.**

(a) Representative images of CD8 and PD-L1 IHC from 3 additional diffuse midline glioma cases.

(b) Representative images of CD3 and CD163 IHC from 3 different DMG cases. Scale bar is 100 μm. Images taken at 100x magnification.

**Figure S9** (related to Fig 5)

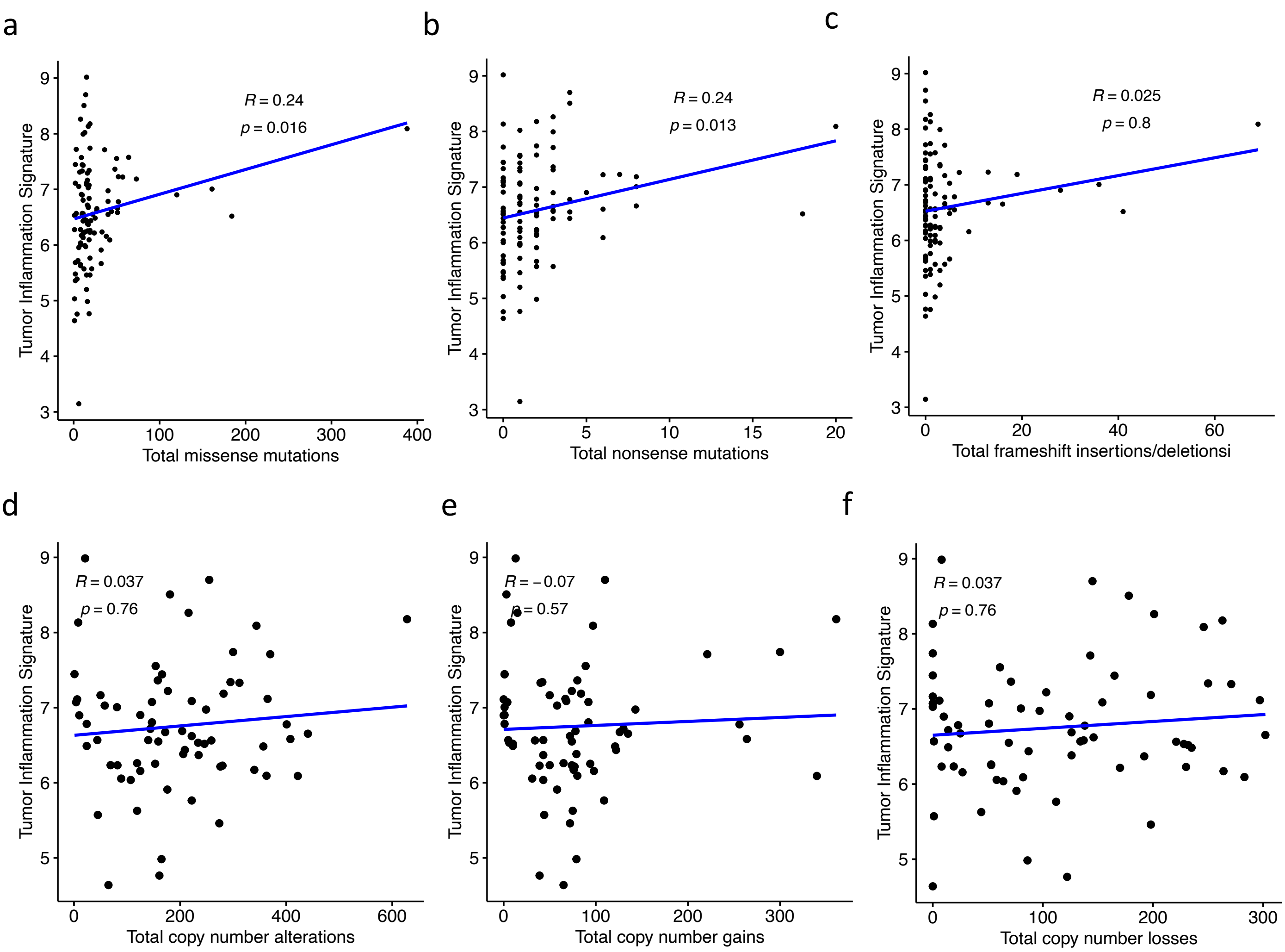

**Figure S9: TIS correlations with genomic features for pediatric HGG.**

Scatter plots of TIS versus (a) total number of missense mutations, (b) total nonsense mutations, (c) total frameshift indels, (d) total number of copy number alterations, (e) copy number gains, (f) copy number losses. N=180 HGGs from PBTA dataset. Correlation coefficient and p-values calculated using Spearman correlation.

**Figure S10** (related to Fig 6)

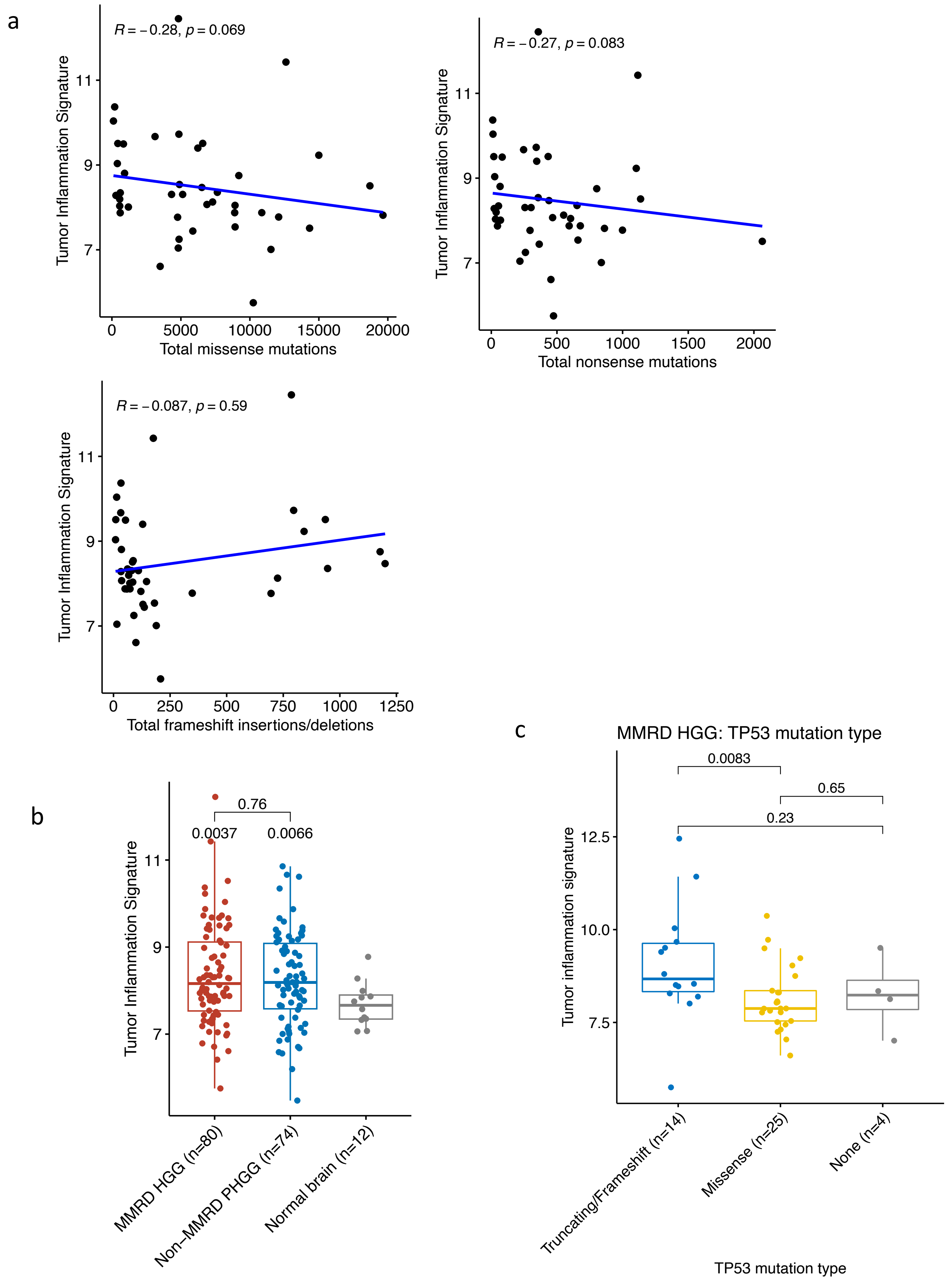

**Figure S10: TIS correlations with genomic features for MMRD HGG.**

(a) Scatter plots comparing TIS to total nonsense mutations, total missense mutations, and total frameshift indels in MMRD HGGs. Correlation coefficient and p-values calculated using Spearman correlation. N=42 samples.

(b) Boxplots comparing TIS in MMRD and non-MMRD HGGs. Boxes show the median and interquartile range (IQR) of the data with whiskers extending to ±1.5 IQR. P-values by two-tailed T-test with Holm adjustment.N=40.

(c) Boxplots of TIS in MMRD HGGs split by type of TP53 mutation (truncating/frameshift, missense, or wild type). P-values by two-tailed Wilcoxon rank sum test.

**Figure S11** (related to Fig 6)

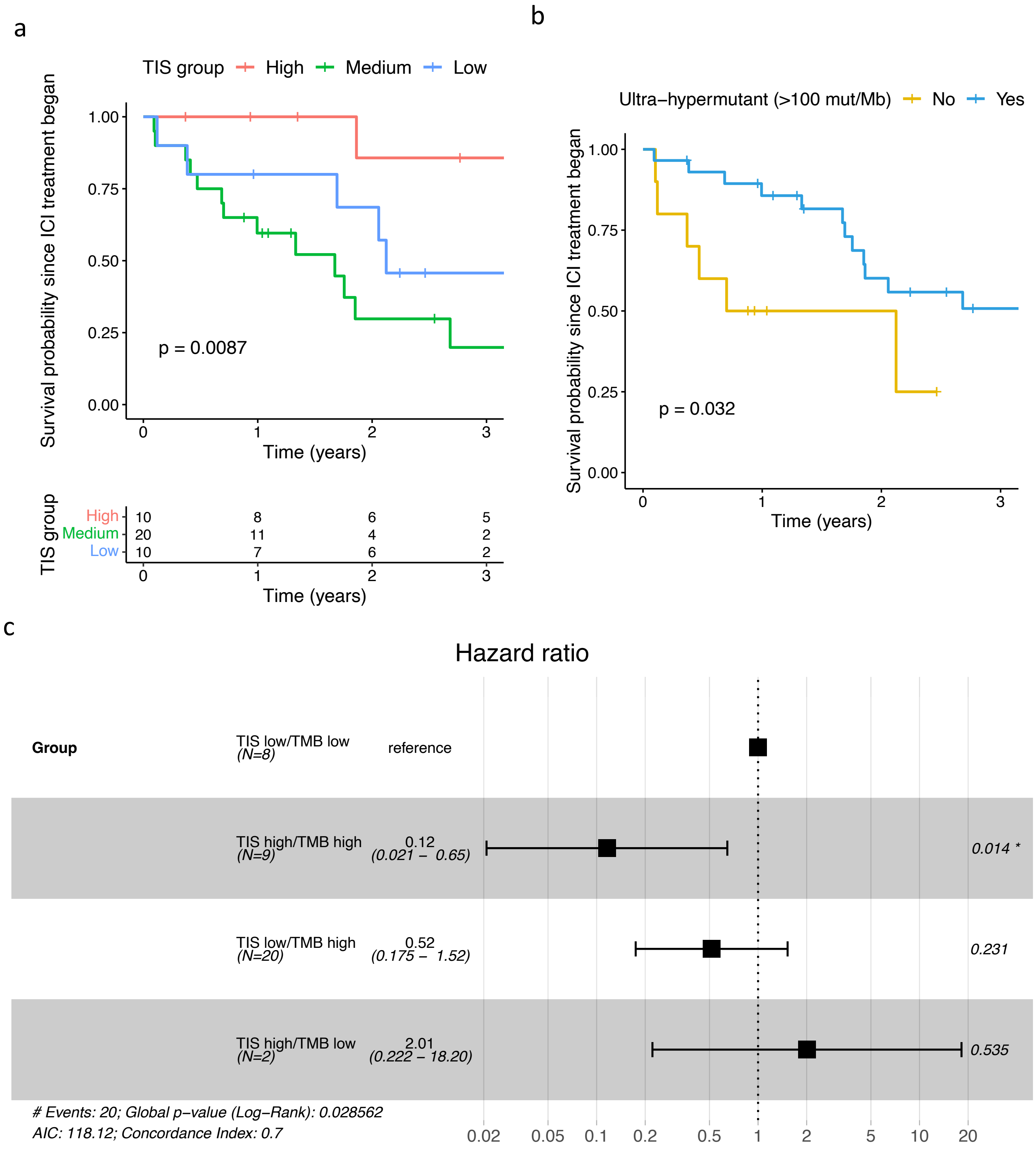

**Figure S11: Additional survival analysis for MMRD HGG.**

(a-b) Kaplan-Meier curve of survival for immune checkpoint inhibitor (ICI) treated MMRD HGG separated by (a) TIS quartiles and (b).ultra-hypermutant (TMB > 100 SNV/Mb) vs non-ultrahypermutant status. P-values by log-rank test.

(c) Forest plot comparing hazard ratios for 4 patient groups split by TIS and TMB levels from Fig 6c. P-values calculated in reference to TIS-low/TMB-low group by log-rank test..

**Figure S12** (related to Fig 6 and 7)

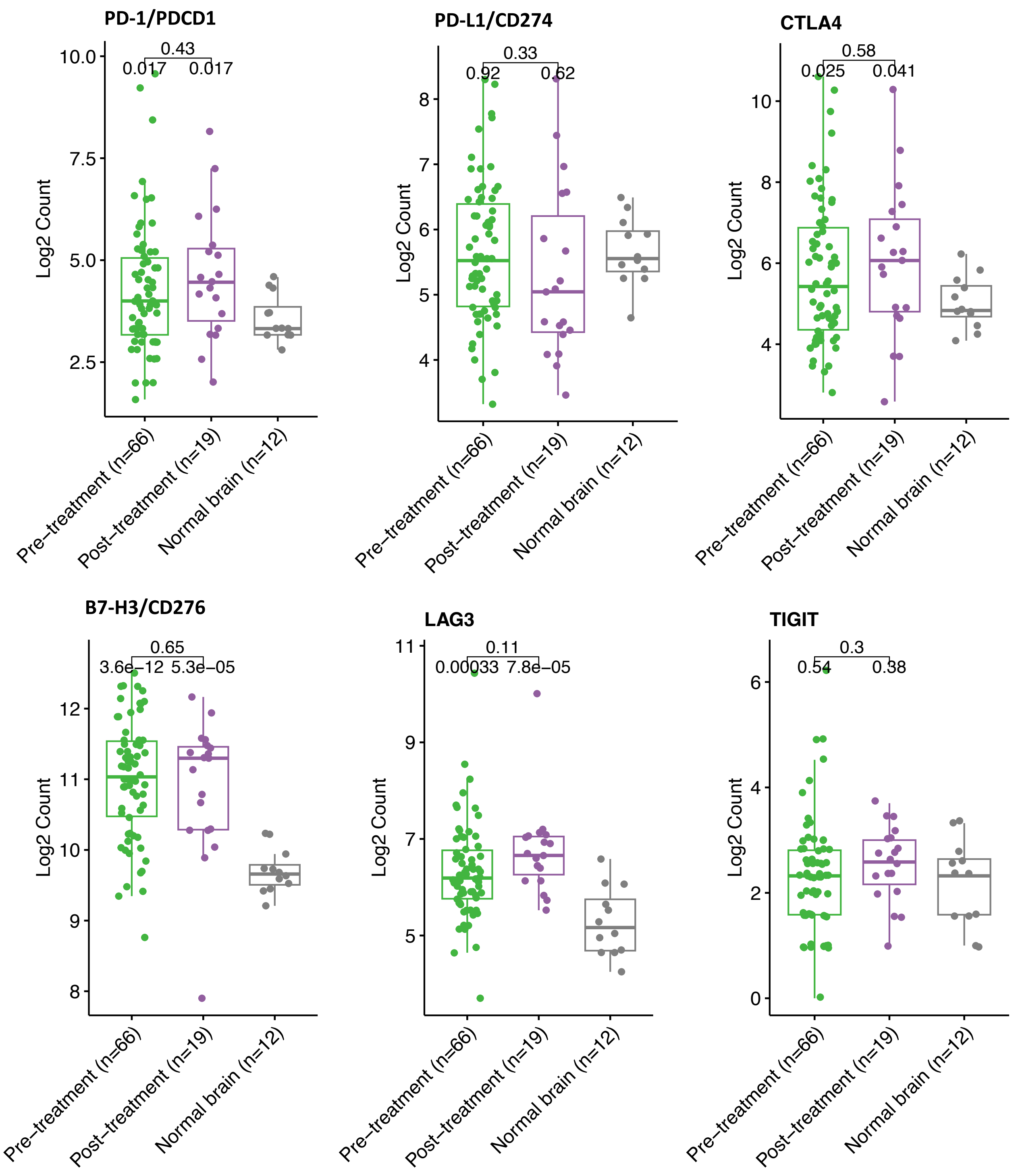

**Figure S12: Gene expression levels for MMRD HGG.**

Boxplots of PD-1, PD-L1, CTLA4, B7-H3 (CD276), LAG3, and TIGIT comparing pre-ICI treatment (or never treated) MMRD HGGs with post-treatment samples. Boxes show the median and interquartile range (IQR) of the data with whiskers extending to ±1.5 IQR. P values by two-tailed T-test with Holm adjustment.

**Figure S13** (related to Fig 7)

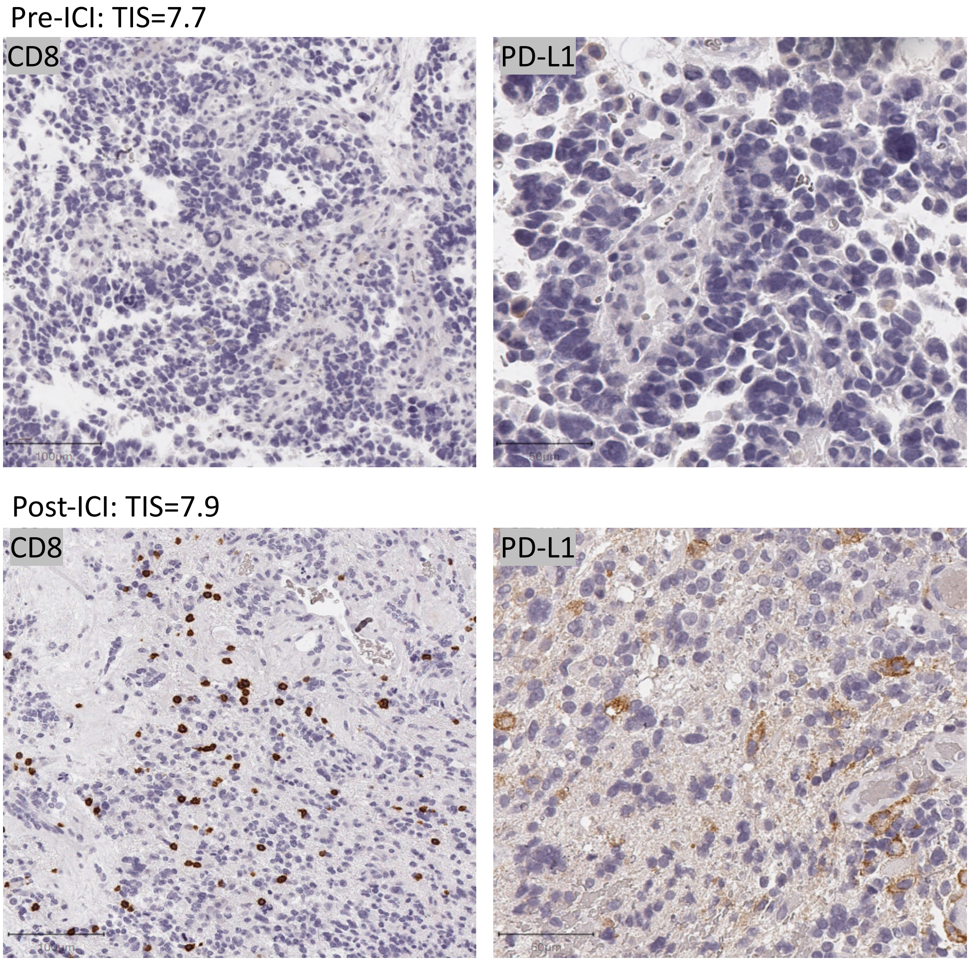

**Figure S13: Additional pre/post-ICI IHC for MMRD HGG.**

Representative images of CD8 and PD-L1 IHC for a second MMRD HGG patient treated with ICI, comparing pre-treatment and post-treatment samples.

# Figure S14

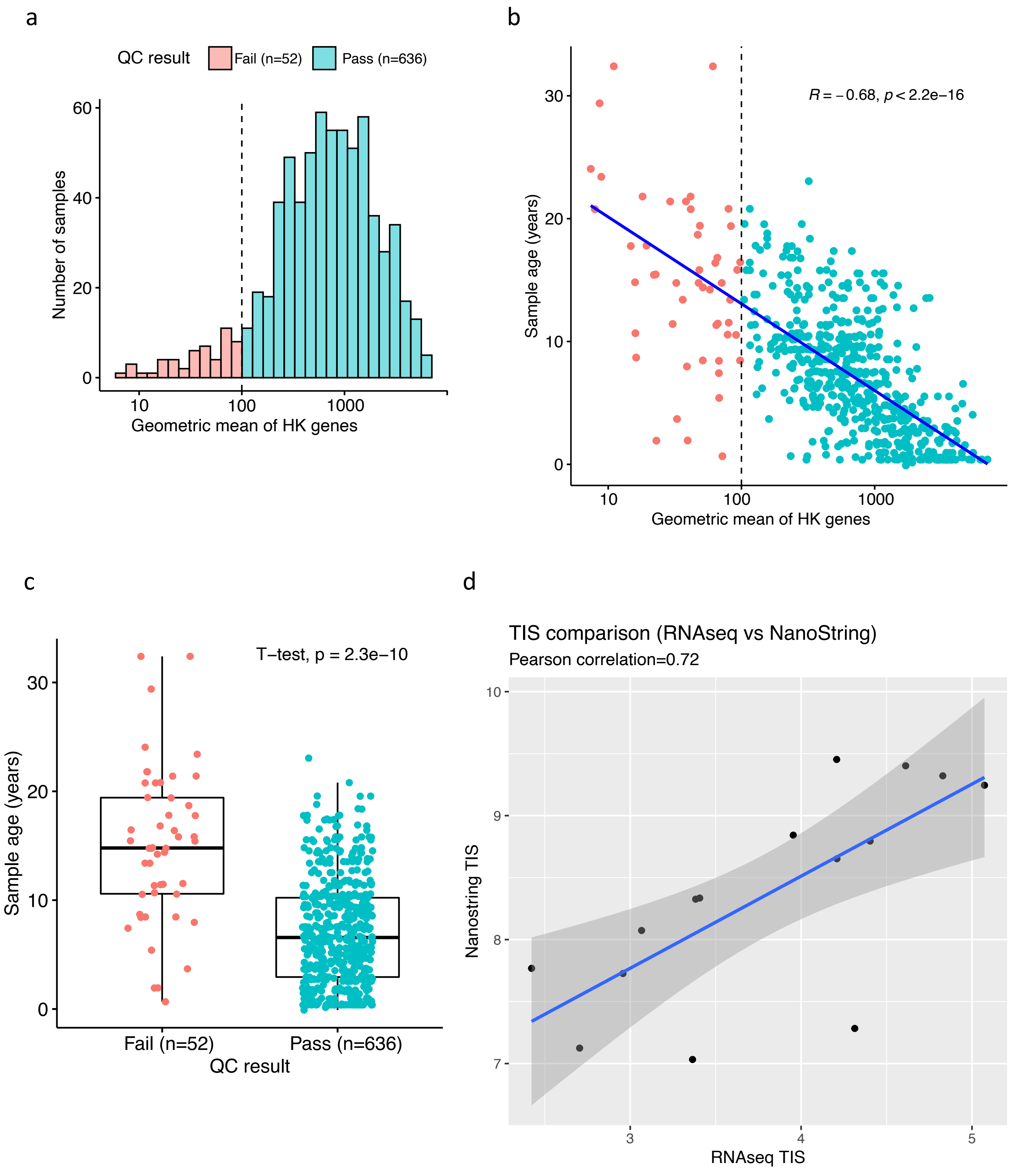
a
QC result
Fail (n=52)
Pass (n=636)
Number of samples
Geometric mean of HK genes
b
R = −0.68, p < 2.2e−16
Sample age (years)
Geometric mean of HK genes
c
T−test, p = 2.3e−10
Sample age (years)
Fail (n=52)
Pass (n=636)
QC result
d
TIS comparison (RNAseq vs NanoString)
Pearson correlation=0.72
Nanostring TIS
RNAseq TIS

**Figure S14: NanoString assay validation and QC data**.

(a) Histogram of housekeeping (HK) gene geometric mean values for 688 samples. The dashed vertical line indicates the QC cutoff of 100.

(b) Scatter plot of sample age vs. HK geometric mean values. P-value calculated using the Spearman coefficient. Total N=688 samples.

(c) Boxplot of sample age for samples that failed and ones that passed the QC metric of a minimum HK geometric mean of 100. Boxes show the median and interquartile range (IQR) of the data with whiskers extending to ±1.5 IQR. P-value by two-tailed T-test.

(d) Correlation of Tumor inflammation Signature (TIS) calculated with NanoString gene counts vs. RNAseq gene counts (TPM) for 16 samples (all high-grade gliomas) with both NanoString and RNAseq available. Error bars show 95% confidence interval.
