# Supplementary material for: Immuno-oncologic profiling of pediatric brain tumors reveals major clinical significance of the tumor immune microenvironment
Source: Nat Commun. 2024 Jul 10;15:5790. doi: 10.1038/s41467-024-49595-1 (PMC11237052; doi:10.1038/s41467-024-49595-1)
Supplement: Supplementary file 3 — Description of Additional Supplementary Files [file 41467_2024_49595_MOESM3_ESM.pdf]

# Description of Additional Supplementary Files

**Supplementary Data 1:** Normalized NanoString counts matrix for full SickKids patient cohort

**Supplementary Data 2:** Immunohistochemistry quantification for comparison with NanoString gene counts

**Supplementary Data 3:** Sample type and subtype for overall SickKids patient cohort

**Supplementary Data 4:** Genes on custom NanoString immuno-oncology panel

**Supplementary Data 5:** Tumor inflammation signature scores for PBTA patient cohort

**Supplementary Data 6:** Immune signature GSVA scores, using gene sets from Bagaev et al, Cancer Cell 2021

**Supplementary Data 7:** PROGENy pathway activation scores for PBTA samples

**Supplementary Data 8:** Pearson correlation values for TIS with COSMIC SBS signatures

**Supplementary Data 9:** Clinical, pathologic, and molecular annotation for SickKids low-grade glioma cohort

**Supplementary Data 10:** Differential expression testing for BRAF mutant vs. BRAF fused LGG

**Supplementary Data 11:** Consensus clusters for BRAF mutant low-grade gliomas

**Supplementary Data 12:** Estimated tumor content for BRAF mutant low-grade gliomas

**Supplementary Data 13:** Clinical and molecular annotations for SickKids high-grade glioma cohort

**Supplementary Data 14:** TIS scores for primary vs progression/recurrent diffuse midline gliomas

**Supplementary Data 15:** SNV effect breakdown for PBTA high-grade gliomas

**Supplementary Data 16:** CNV breakdown for PBTA high-grade gliomas

**Supplementary Data 17:** Clinical data for MMRD HGG patient cohort

**Supplementary Data 18:** Outcome data and tumor mutation burden for immune checkpoint inhibitor treated MMRD HGGs

**Supplementary Data 19:** Breakdown of variants types from WES data for MMRD HGGs

**Supplementary Data 20:** TP53 variant types for MMRD HGGs

**Supplementary Data 21:** Sample QC metrics and pass/fail status compared to sample age

**Supplementary Data 22:** NanoString vs RNAseq TIS scores for validation samples
