# Supplementary material for: Immuno-oncologic profiling of pediatric brain tumors reveals major clinical significance of the tumor immune microenvironment
Source: Nat Commun. 2024 Jul 10;15:5790. doi: 10.1038/s41467-024-49595-1 (PMC11237052; doi:10.1038/s41467-024-49595-1)
Supplement: Supplementary file 5 — Reporting Summary [file 41467_2024_49595_MOESM5_ESM.pdf]

nature portfolio

Corresponding author(s): Cynthia Hawkins
Uri Tabori

Last updated by author(s): Feb 19, 2024

Reporting Summary

Nature Portfolio wishes to improve the reproducibility of the work that we publish. This form provides structure for consistency and transparency in reporting. For further information on Nature Portfolio policies, see our Editorial Policies and the Editorial Policy Checklist.

Statistics

For all statistical analyses, confirm that the following items are present in the figure legend, table legend, main text, or Methods section.

n/a	Confirmed	
☐	☒	The exact sample size (*n*) for each experimental group/condition, given as a discrete number and unit of measurement
☐	☒	A statement on whether measurements were taken from distinct samples or whether the same sample was measured repeatedly
☐	☒	The statistical test(s) used AND whether they are one- or two-sided<br>*Only common tests should be described solely by name; describe more complex techniques in the Methods section.*
☐	☒	A description of all covariates tested
☐	☒	A description of any assumptions or corrections, such as tests of normality and adjustment for multiple comparisons
☐	☒	A full description of the statistical parameters including central tendency (e.g. means) or other basic estimates (e.g. regression coefficient) AND variation (e.g. standard deviation) or associated estimates of uncertainty (e.g. confidence intervals)
☐	☒	For null hypothesis testing, the test statistic (e.g. *F*, *t*, *r*) with confidence intervals, effect sizes, degrees of freedom and *P* value noted<br>*Give P values as exact values whenever suitable.*
☒	☐	For Bayesian analysis, information on the choice of priors and Markov chain Monte Carlo settings
☒	☐	For hierarchical and complex designs, identification of the appropriate level for tests and full reporting of outcomes
☐	☒	Estimates of effect sizes (e.g. Cohen's *d*, Pearson's *r*), indicating how they were calculated

Our web collection on statistics for biologists contains articles on many of the points above.

Software and code

Policy information about availability of computer code

Data collection	Microsoft Excel v16.71 and FileMaker Pro Advanced v18 were used for clinical data collection.
Data analysis	R version 4.1 and 4.2 with Bioconductor 3.14 and 3.16 were used with the following packages: tidyverse (v1.3.1) NanoStringQCPro (v1.26.0), ComplexHeatmap (v2.10.1), ConsensusClusterPlus (v1.58.0), genefilter (v1.76.0), tximport (v1.22.0), progeny (v1.20.0), GSVA (v1.46.0), ggpubr (v0.4.0), survival (v3.4-0) survminer (v0.4.9), survivalAnalysis (v0.3.0) .<br>For sequencing data, Mutect2 (GATK v4.2.3.0), Funcotator (GATK v4.2.3.0), DRAGEN (v3.2.8), and Arriba (v2.4.0) were used.<br>Example scripts for processing NanoString data, including normalization and TIS score calculation, are available on GitHub at: https://github.com/adrianblevine/Immuno-oncology_nanostring.

For manuscripts utilizing custom algorithms or software that are central to the research but not yet described in published literature, software must be made available to editors and reviewers. We strongly encourage code deposition in a community repository (e.g. GitHub). See the Nature Portfolio guidelines for submitting code & software for further information.

Data

Policy information about availability of data

All manuscripts must include a data availability statement. This statement should provide the following information, where applicable:

- Accession codes, unique identifiers, or web links for publicly available datasets
- A description of any restrictions on data availability
- For clinical datasets or third party data, please ensure that the statement adheres to our policy

The NanoString data newly generated on our cohort is available at GEO under GSE227756 [https://www.ncbi.nlm.nih.gov/geo/query/acc.cgi?acc=GSE227756]. Newly generated panel sequencing data of high-grade gliomas is available at EGA under study EGAS50000000221 and dataset EGAD50000000326 [https://ega-archive.org/datasets/EGAD50000000326]. The data are available under controlled access to comply with data protection regulations and can be accessed by application to the data access committee via C.H. (cynthia.hawkins@sickkids.ca). No specific restrictions are in place for granting access to the data, other than a data transfer agreement to ensure academic use of the data. Data will be available for 1 year once granted, although extensions can be made if needed. The remaining data, including normalized NanoString gene counts, are available within the Source Data file with the paper.

Previously published WES data from tumors with matched germline blood for MMRD patients treated with immune checkpoint inhibition8 is available on EGA under EGAD00001008036 [https://ega-archive.org/datasets/EGAD00001008036].This can accessed by application to the data access committee via U.T. (uri.tabori@sickkids.ca). Previously published RNA and targeted DNA sequencing data for PLGG53 is available on EGA under EGAD00001005987 [https://ega-archive.org/datasets/EGAD00001005987] and can be accessed by application to the data access committee via C.H. (cynthia.hawkins@sickkids.ca). Additional clinical data and molecular characterization (using IHC, FISH, NanoString gene fusion panels, SNP array) is available as source data at the manuscript website https://doi.org/10.1016/j.ccell.2020.03.011. Previously published sequencing data for DMG and hemispheric PHGG63–65 is available on EGA as follows:
WGS: EGAD00001000814 [https://ega-archive.org/datasets/EGAD00001000814], EGAD00001003305 [https://ega-archive.org/datasets/EGAD00001003305],
WES: EGAD00001006450 [https://ega-archive.org/datasets/EGAD00001006450], EGAD00001008279 [https://ega-archive.org/datasets/EGAD00001008279], EGAD00001003305 [https://ega-archive.org/datasets/EGAD00001003305]
RNA-seq: EGAD00001006450 [https://ega-archive.org/datasets/EGAD00001006450], EGAD00001008278 [https://ega-archive.org/datasets/EGAD00001008278]
These can be can be accessed by application to the data access committee via C.H. (cynthia.hawkins@sickkids.ca).

The publicly available PBTA raw data is available through KidsFirstPortal (https://portal.kidsfirstdrc.org/login) accession codes PBTA-CBTN and PBTA-PNOC. and Cavatica (https://cavatica.sbgenomics.com/u/cavatica/openpbta) upon request to CBTN, and processed summary files are accessible at https://github.com/AlexsLemonade/OpenPBTA-analysis.

Research involving human participants, their data, or biological material

Policy information about studies with human participants or human data. See also policy information about sex, gender (identity/presentation), and sexual orientation and race, ethnicity and racism.

Reporting on sex and gender	Sex and gender were not considered in the study design. Sex based analysis was not performed, as the focus of this study was on the tumor immune microenvironment across pediatric brain tumor types. For patients where it is available in the clinical databases, the sex is reported in the relevant supplemental tables.
Reporting on race, ethnicity, or other socially relevant groupings	Race, ethnicity, and other socially relevant groupings were not considered in the study design, as the focus of this study was on the tumor immune microenvironment across pediatric brain tumor types.
Population characteristics	Samples were taken from fully-consented patients diagnosed with pediatric brain tumors at the Hospital for Sick Children, ages 0-18 years, from diverse ethnic backgrounds. For comparison with an adult population, samples from our Toronto-wide adult and young adolescent (AYA) brain tumor patient cohort were used, which includes patients aged 15-40 years.
Recruitment	Written consent from a legally authorised representative was obtained to collect tissue for research in all autopsy cases and in all surgical cases collected since 2010, with explicit consent for use for next generation sequencing since 2016. For surgical cases prior to 2010 or where the consent did not explicitly state the tissue would be used for next generation sequencing, for deceased patients, waiver of consent to use the tissue for this purpose was granted by the Hospital for Sick Children Research Ethics Board. After obtaining consent, tumour and normal brain samples were taken at surgery or postmortem from pediatric patients diagnosed with a brain tumor presenting to The Hospital for Sick Children and immediately snap frozen in liquid nitrogen and stored at -80 C, or stored as formalin-fixed paraffin embedded tissue (FFPE).
Ethics oversight	The Hospital for Sick Children Research Ethics Board provided ethical oversight (#1000055059). Informed consent was provided for all participants.

Note that full information on the approval of the study protocol must also be provided in the manuscript.

Field-specific reporting

Please select the one below that is the best fit for your research. If you are not sure, read the appropriate sections before making your selection.

☒ Life sciences ☐ Behavioural & social sciences ☐ Ecological, evolutionary & environmental sciences

For a reference copy of the document with all sections, see nature.com/documents/nr-reporting-summary-flat.pdf

Life sciences study design

All studies must disclose on these points even when the disclosure is negative.

Sample size	No statistical methods were used to pre-determine sample sizes. Sample sized was based on the number of samples of tumor types of interest with available tissue for profiling.
Data exclusions	No data were excluded from analysis, other than those failing QC metrics for NanoString and other molecular testing, as described in the Methods section.
Replication	Accuracy and reproducibility of the results from the NanoString immuno-oncology panel were tested during the clinical panel validation, including comparison with orthogonal gene expression profiling methods, as described in the Methods section. Intra-run (3 replicates in same run) and inter-run (3 replicates in different runs) reproducibility was evaluated using 4 samples. All attempts at replication were successful. Findings from the SickKids patient cohort with respect to the overall patterns of inflammation in different tumor types were replicated in the external PBTA patient cohort, with the exception of some rare patient populations (e.g. mismatch repair deficient gliomas) that are not sufficiently represented in the PBTA data to permit analysis.
Randomization	Randomization was not applied because we studied differences between specimens of known origins, e.g. tumors with different classifications.
Blinding	We did not blind as the primary objective was to compare profiles between different tumor types, which required knowledge of the specific tumor classifications.

Reporting for specific materials, systems and methods

We require information from authors about some types of materials, experimental systems and methods used in many studies. Here, indicate whether each material, system or method listed is relevant to your study. If you are not sure if a list item applies to your research, read the appropriate section before selecting a response.

Materials & experimental systems

n/a	Involved in the study	
☐	☒	Antibodies
☒	☐	Eukaryotic cell lines
☒	☐	Palaeontology and archaeology
☒	☐	Animals and other organisms
☐	☒	Clinical data
☒	☐	Dual use research of concern
☒	☐	Plants

Methods

n/a	Involved in the study	
☒	☐	ChIP-seq
☒	☐	Flow cytometry
☒	☐	MRI-based neuroimaging

Antibodies

Antibodies used	- PD-L1 (clone:28-8, Rabbit monoclonal Abcam, 1:500 cat no: ab205921)<br>- CD68 (clone:PG-M1, Dako-Omnis, ready to use, cat no GA613)<br>- CD8 (clone:c8/144B, Dako-Omnis, ready to use, cat no:GA623)<br>- CD3 (polyclonal rabbit, Dako-Omnis, ready to use, cat no:GA503)<br><br>IHCs were done in real-time, often for clinical indications, over the course of several years, in the CLIA approved pathology laboratory at the Hospital for Sick Children. The lot numbers varied according to the time an individual case was stained and hence cannot be individually detailed here.
Validation	All antibodies used are commercially available and were validated by the manufacturer with the details available on their respective websites:<br>- PD-L1: https://www.abcam.com/products/primary-antibodies/pd-l1-antibody-28-8-ab205921.html<br>- CD68: https://www.agilent.com/en/product/immunohistochemistry/antibodies-controls/primary-antibodies/cd68-%28dako-omnis%29-76223<br>- CD8: https://www.agilent.com/en/product/immunohistochemistry/antibodies-controls/primary-antibodies/cd8-%28dako-omnis%29-76236<br>- CD3: https://www.agilent.com/en/product/immunohistochemistry/antibodies-controls/primary-antibodies/cd3-(dako-omnis)-76197

Clinical data

Policy information about clinical studies

All manuscripts should comply with the ICMJE guidelines for publication of clinical research and a completed CONSORT checklist must be included with all submissions.

Clinical trial registration	NCT02992964
Study protocol	https://clinicaltrials.gov/study/NCT02992964
Data collection	This was an investigator-initiated, multicenter, open-label, single-arm pilot study in which pediatric patients ages ≥12 months and <25 years of age with relapsed/refractory cancers with elevated TMB and/or MMRD were treated with nivolumab 3 mg/kg every 2 weeks until confirmed disease progression, intolerable toxicity, or for a maximum of 24 months. The trial was open from 2017-05-15 to 2023-11-20.
Outcomes	The previously published (PMID 37126021) primary endpoint was objective response rate by iRANO (immunotherapy response assessement in neuro-oncology) criteria; secondary endpoints were overall survival, progression free survival, and safety/toxicity of treatment. Analyses performed here represent non-prespecified exploratory outcomes for NCT02992964

Plants

Seed stocks	unable to remove this section
Novel plant genotypes	unable to remove this section
Authentication	unable to remove this section
